# Sensory neuron BRAF mediates opioid-induced hyperalgesia and tolerance via presynaptic NMDA receptor hyperactivity

**DOI:** 10.1172/JCI201808

**Published:** 2026-07-14

**Authors:** Daozhong Jin, Hong Chen, Yuying Huang, Shao-Rui Chen, Hui-Lin Pan

**Affiliations:** Center for Neuroscience and Pain Research, Department of Anesthesiology and Perioperative Medicine, The University of Texas MD Anderson Cancer Center, Houston, Texas, USA.

**Keywords:** Neuroscience, Oncology, Drug therapy, Neurological disorders

## Abstract

Opioids are essential analgesics for managing severe pain but can paradoxically increase pain sensitivity (hyperalgesia) and diminish analgesic efficacy (tolerance). Hyperactivity of NMDA-type glutamate receptors (NMDARs) at primary afferent terminals in the spinal cord contributes to both phenomena; however, the underlying signaling mechanisms remain unclear. Here, we report that morphine administration in rats promoted the translocation of monomeric BRAF, an oncogenic kinase, from the dorsal root ganglion (DRG) to spinal cord synaptosomes, leading to increased MEK-ERK phosphorylation at nociceptor central terminals. BRAF physically interacted with NMDARs in both rat and human spinal cords. Inhibition of BRAF activity with vemurafenib reversed morphine-induced NMDAR phosphorylation and synaptic localization of α2δ-1–bound NMDARs. Vemurafenib also abolished morphine-induced presynaptic NMDAR hyperactivity in spinal dorsal horn neurons. Correspondingly, conditional *Braf* knockout in DRG neurons normalized morphine-enhanced NMDAR phosphorylation, synaptic trafficking of α2δ-1–bound NMDARs, and NMDAR hyperactivity in the spinal cord. Furthermore, pharmacological inhibition of BRAF or MEK, or *Braf* deletion in DRG neurons, enhanced morphine analgesia while mitigating morphine-induced hyperalgesia and tolerance. These findings identify BRAF overactivity at nociceptor central terminals as a key mediator of opioid-induced NMDAR hyperactivity. Clinically approved BRAF inhibitors could be repurposed to enhance opioid analgesia while minimizing adverse effects.

## Abstract 2

Opioids activate a cancer-related protein kinase that increases pain signaling. Blocking this kinase improved analgesic efficacy and reduced opioid-induced tolerance and abnormal pain signaling.

## Graphical abstract

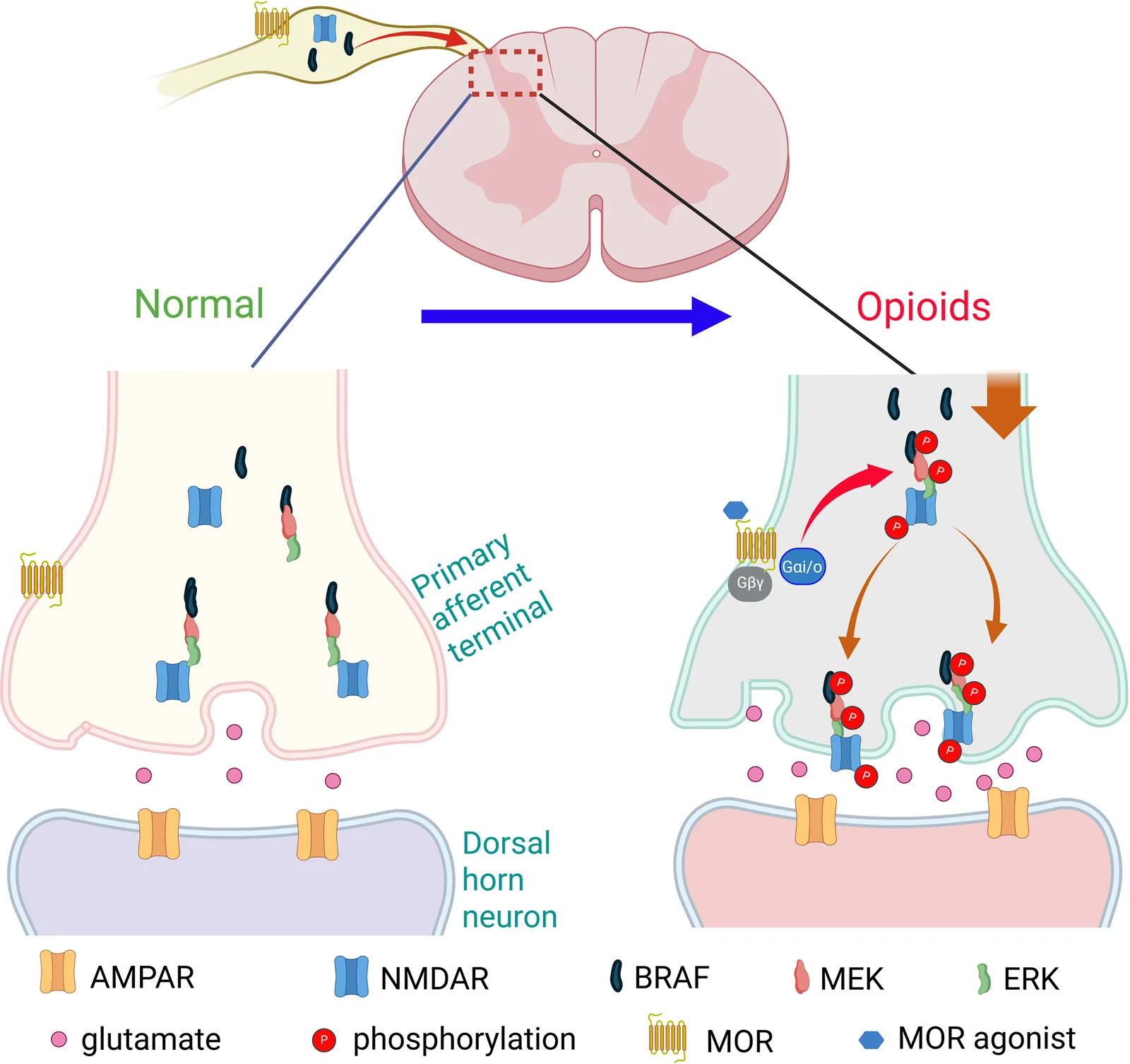

## Introduction

Opioids remain the most potent and indispensable analgesics for managing moderate to severe pain resulting from major surgery, trauma, and cancer. Paradoxically, opioid treatment can lead to a progressive increase in pain sensitivity (hyperalgesia) alongside a reduction in analgesic efficacy (tolerance) (1–3). Notably, even acute exposure to opioids such as morphine and fentanyl can produce long-lasting hyperalgesia in both animal models and humans (4–8). These maladaptive responses often drive unsafe dose escalation, increasing the risk of dependence, addiction, and overdose death. A major challenge in pain control is identifying the signaling pathways that mediate the shift in opioid effects from analgesia to hyperalgesia in vivo. μ-Opioid receptors (MORs), which mediate opioid analgesia, are abundantly expressed in dorsal root ganglion (DRG) neurons and their central terminals in the spinal dorsal horn (9, 10). Targeted deletion of MORs in DRG neurons eliminates both the inhibitory and excitatory effects of opioids on spinal nociceptive transmission, thereby diminishing the analgesic and hyperalgesic effects of systemically administered opioids (11–13). These findings highlight the critical importance of elucidating the signaling mechanisms operating at sensory neuron–dorsal horn neuron synapses that govern the shift in opioid actions from inhibition to excitation.

MOR activation induces sustained hyperactivity of N-methyl-D-aspartate receptors (NMDARs) at primary afferent terminals in the spinal cord, playing a crucial role in opioid-induced hyperalgesia and tolerance (1, 3, 12, 14–16). NMDARs at primary afferent terminals in the spinal dorsal horn are normally quiescent due to intracellular retention by LRRC8A proteins (17). However, short- and long-term use of opioids promotes NMDAR synaptic trafficking and hyperactivity, amplifying nociceptive transmission (3, 12, 16). NMDAR phosphorylation is a key event driving this synaptic localization and hyperactivity in the spinal cord (3, 18–20). This phosphorylation is typically mediated by protein kinase C (PKC) through cross-talk between MORs and Gαq signaling (3, 20). Additionally, opioids activate extracellular signal-regulated kinase (ERK) in cultured cells transfected with MORs (21–26), and ERK activation contributes to NMDAR hyperactivity in both the brain and spinal cord (18, 27). Pharmacological inhibition of ERK reduces opioid-induced NMDAR hyperactivity, hyperalgesia, and tolerance (18). However, the mechanisms by which opioids activate ERK remain unclear. Currently, neither PKC nor ERK inhibitors are used clinically due to intolerable adverse effects, underscoring the need for alternative, clinically viable kinase targets to mitigate opioid-induced hyperalgesia and tolerance.

RAF (rapidly accelerated fibrosarcoma) kinases are serine/threonine-specific enzymes that function upstream of mitogen-activated protein kinase kinases (MEK) and ERK signaling pathway. The RAF family comprises three isoforms: ARAF, BRAF, and CRAF (also known as RAF-1). Notably, BRAF, but not CRAF, regulates ERK phosphorylation in neurons (28). BRAF is the only RAF kinase highly enriched at synapses and distributed throughout neuronal somas and processes (29), whereas CRAF is largely confined to the perinuclear cytosol (30). Although BRAF is most recognized for its role in oncogenesis, particularly through activating mutations like BRAF^V600E^ (31, 32), its synaptic localization and involvement in G-protein signaling (29, 33, 34) suggest that it may serve as a critical link between MOR activation and synaptic NMDAR phosphorylation and hyperactivity. However, its neuronal and synaptic functions remain largely unexplored.

In this study, we investigated whether BRAF signaling contributes to opioid-induced NMDAR phosphorylation and hyperactivity at spinal sensory synapses. We demonstrated that opioid treatment promoted BRAF recruitment to primary afferent terminals in the spinal cord, thereby increasing its activity through MEK-ERK signaling. BRAF inhibition or genetic ablation in DRG neurons attenuated opioid-induced hyperalgesia and tolerance by suppressing NMDAR phosphorylation, synaptic localization, and hyperactivity at nociceptor central terminals. By identifying overactive BRAF as a key mediator of opioid-induced nociceptive plasticity, our findings suggest that BRAF inhibitors currently used in oncology could be repurposed to improve opioid analgesic efficacy while minimizing adverse effects.

## Results

### BRAF is expressed in DRG neuron subtypes and spinal cord.

**Figure 1:**
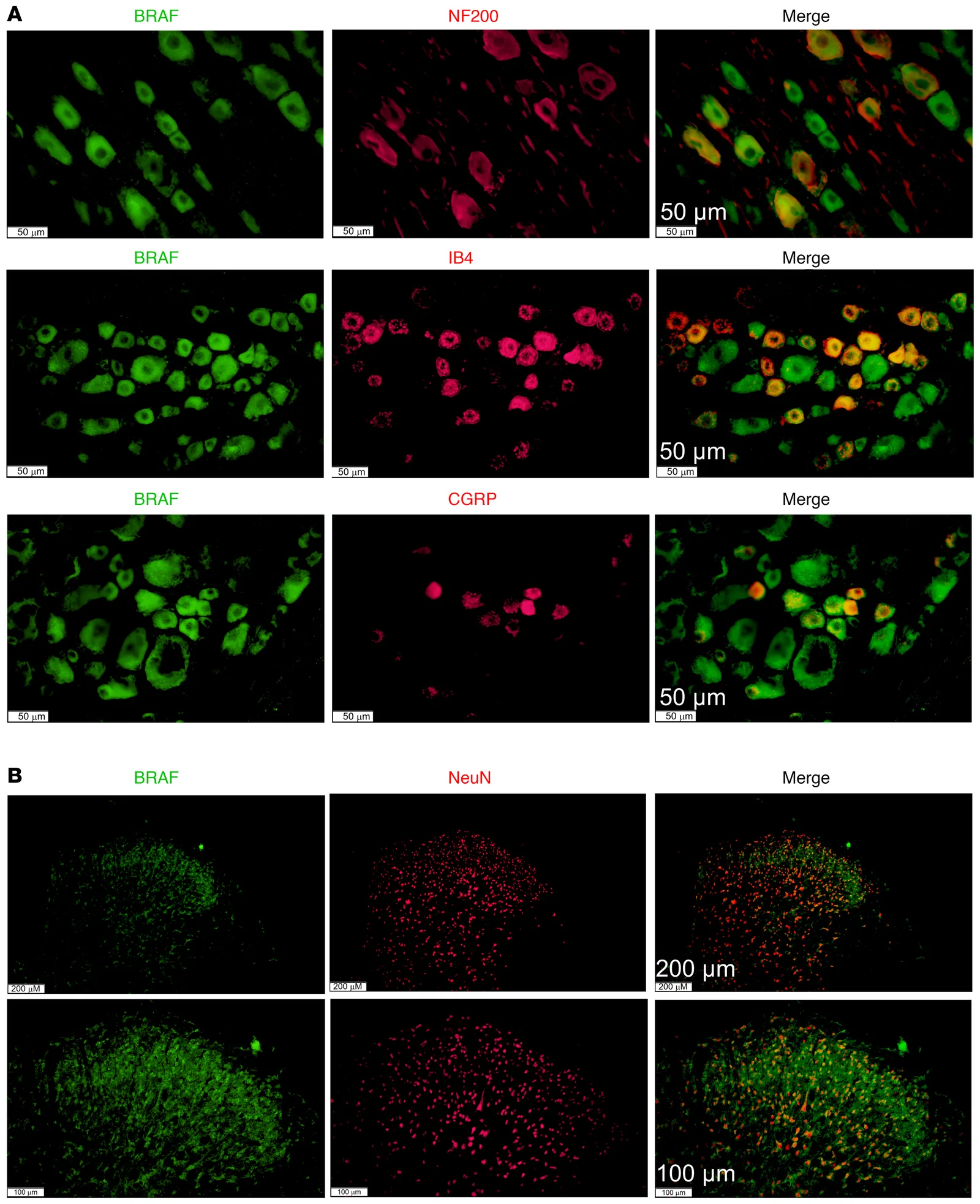
Spatial distribution of BRAF in DRG neuron subtypes and spinal dorsal horn. (**A**) Representative immunofluorescence images show colocalization of BRAF (green) with NF200, IB4, or CGRP (red) in the rat DRG. (**B**) Representative immunofluorescence images show colocalization of BRAF (green) with NeuN (red) in the rat spinal dorsal horn. Scale bar: 50 (**A**), 100 (**B**, bottom row), or 200 (**B**, top row) μm.

BRAF is present in the DRG and spinal cord (28, 35). To characterize the cellular distribution of BRAF, we performed double immunofluorescence labeling in the rat DRG and spinal dorsal horn. Immunofluorescence analysis revealed that BRAF immunoreactivity in the DRG colocalized with NF200, CGRP, and IB4 (Figure 1A), markers of large-diameter neurons, small-diameter peptidergic neurons, and small-diameter nonpeptidergic neurons, respectively. Among BRAF-positive neurons, approximately 35% coexpressed NF200 (38.35 ± 3.46%) or IB4 (33.50 ± 2.04%), whereas about 28% were CGRP-positive (28.16 ± 3.60%). In the spinal cord, BRAF immunoreactivity was distributed throughout the dorsal horn, with particularly dense labeling in laminae I and II. Colocalization with the neuronal marker NeuN confirmed that BRAF was expressed in neurons of the spinal dorsal horn (Figure 1B).

### Opioid treatment promotes BRAF protein translocation from the DRG to the spinal cord.

**Figure 2:**
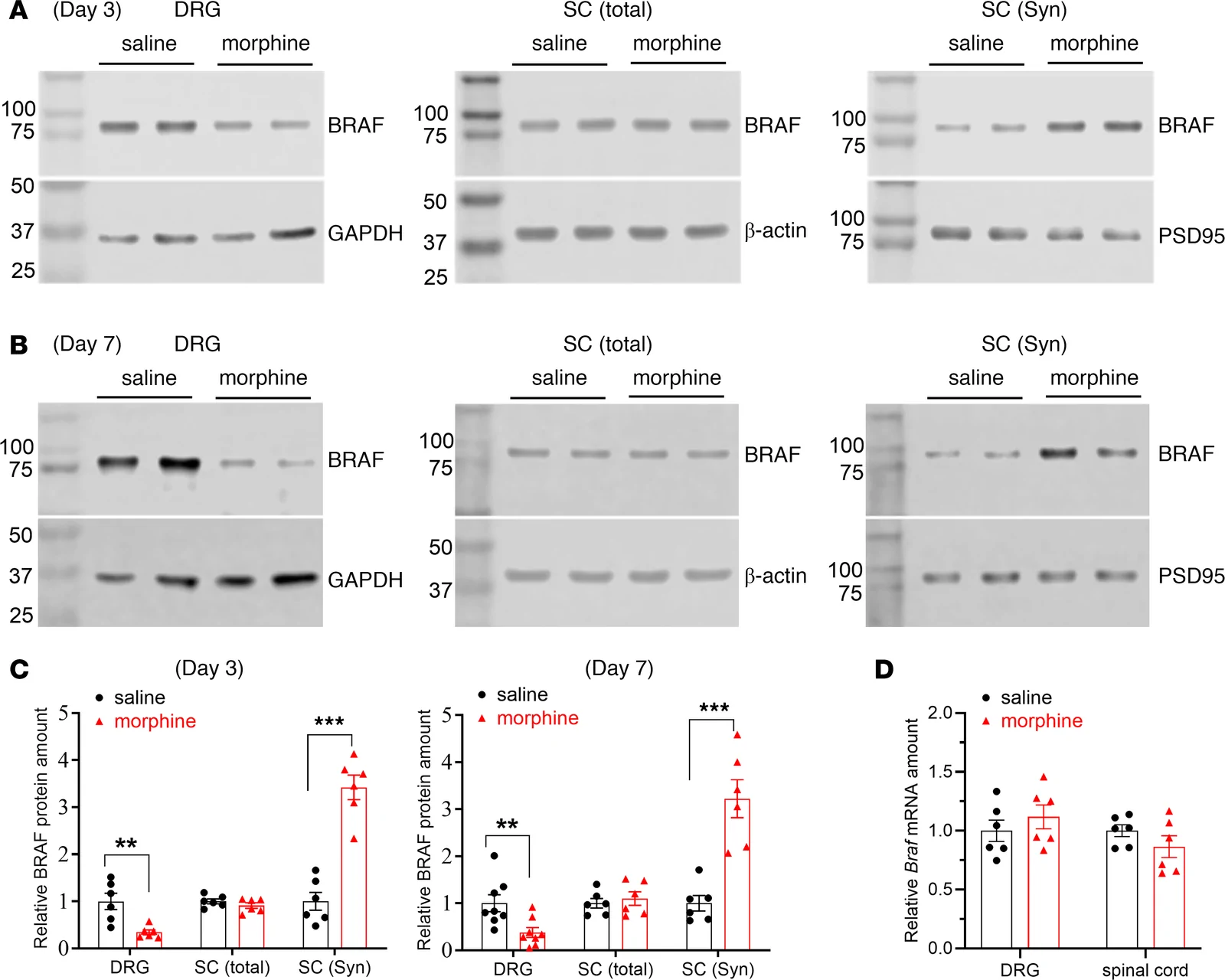
Morphine treatment promotes BRAF protein trafficking from the DRG to spinal cord. (A–C) Representative immunoblot images (**A, B**) and quantification (**C**) show the effect of morphine treatment on the total and synaptosomal (Syn) BRAF protein amount in the DRG and spinal cord (SC). Total and synaptosomal proteins were extracted for immunoblotting analysis (*n* = 6–8 rats per group). GAPDH, β-actin, and PSD95 were used as loading controls on the same membranes. The lumbar DRG and dorsal spinal cord were collected from rats treated with morphine (5 mg/kg) or saline twice daily for 3 or 7 days. **(D)** Real-time PCR analysis shows the effect of morphine treatment on *Braf* mRNA expression in the DRG and spinal cord. Total RNA was extracted for real-time PCR analysis, with β-actin as the reference control (*n* = 6 rats per group). Data are expressed as means ± SEM. **P < 0.01, ***P < 0.001 (2-tailed Student’s t test).

BRAF is a critical kinase in the MAPK/ERK pathway, and its constitutive activation in nonneuronal cells drives cancer progression by amplifying growth-promoting signaling (36, 37). To determine whether opioid treatment influences BRAF expression, lumbar DRG and dorsal spinal cord tissues were collected from rats treated with saline or morphine (5 mg/kg, i.p., twice daily) for 3 or 7 consecutive days. Immunoblotting analysis detected a single BRAF protein band (approximately 85 kDa) in both tissues. Remarkably, morphine treatment for either 3 or 7 days substantially reduced BRAF protein abundance in the DRG compared with saline-treated controls (Figure 2, A–C). In contrast, total BRAF expression in the spinal cord was unchanged; however, BRAF abundance was markedly increased in the synaptosomal fraction following 3 or 7 days of morphine treatment (Figure 2, A–C). Real-time PCR analysis showed that morphine treatment for 7 days did not alter *Braf* mRNA levels in either the DRG or spinal cord (Figure 2D).

To determine whether MOR activation is required for morphine-induced BRAF translocation from the DRG to the spinal cord, rats received a single intrathecal treatment with β-funaltrexamine (FNA, 10 μg), an irreversible MOR antagonist (38), or vehicle followed by morphine treatment (5 mg/kg, i.p., twice daily) for 7 days. As expected, repeated morphine administration in vehicle-treated rats induced mechanical and thermal hyperalgesia as well as analgesic tolerance, evidenced by a progressive reduction in baseline withdrawal thresholds and latencies and a gradual decline in morphine analgesic efficacy. Pretreatment with FNA diminished the development of morphine-induced hyperalgesia and tolerance (Supplemental Figure 1A; supplemental material available online with this article; https://doi.org/10.1172/JCI201808DS1). Moreover, acute morphine injection (5 mg/kg, i.p.) failed to produce analgesic effects on either day 1 or day 7 in FNA-treated rats (Supplemental Figure 1B). Importantly, FNA pretreatment also blocked morphine-induced changes in BRAF expression in the DRG and spinal synaptosomal fractions (Supplemental Figure 1C). Together, these data indicate that morphine treatment promotes MOR-dependent anterograde trafficking of BRAF from DRG neurons to their central terminals in the spinal cord.

### Morphine treatment increases BRAF activity in the spinal cord but not DRG.

**Figure 3:**
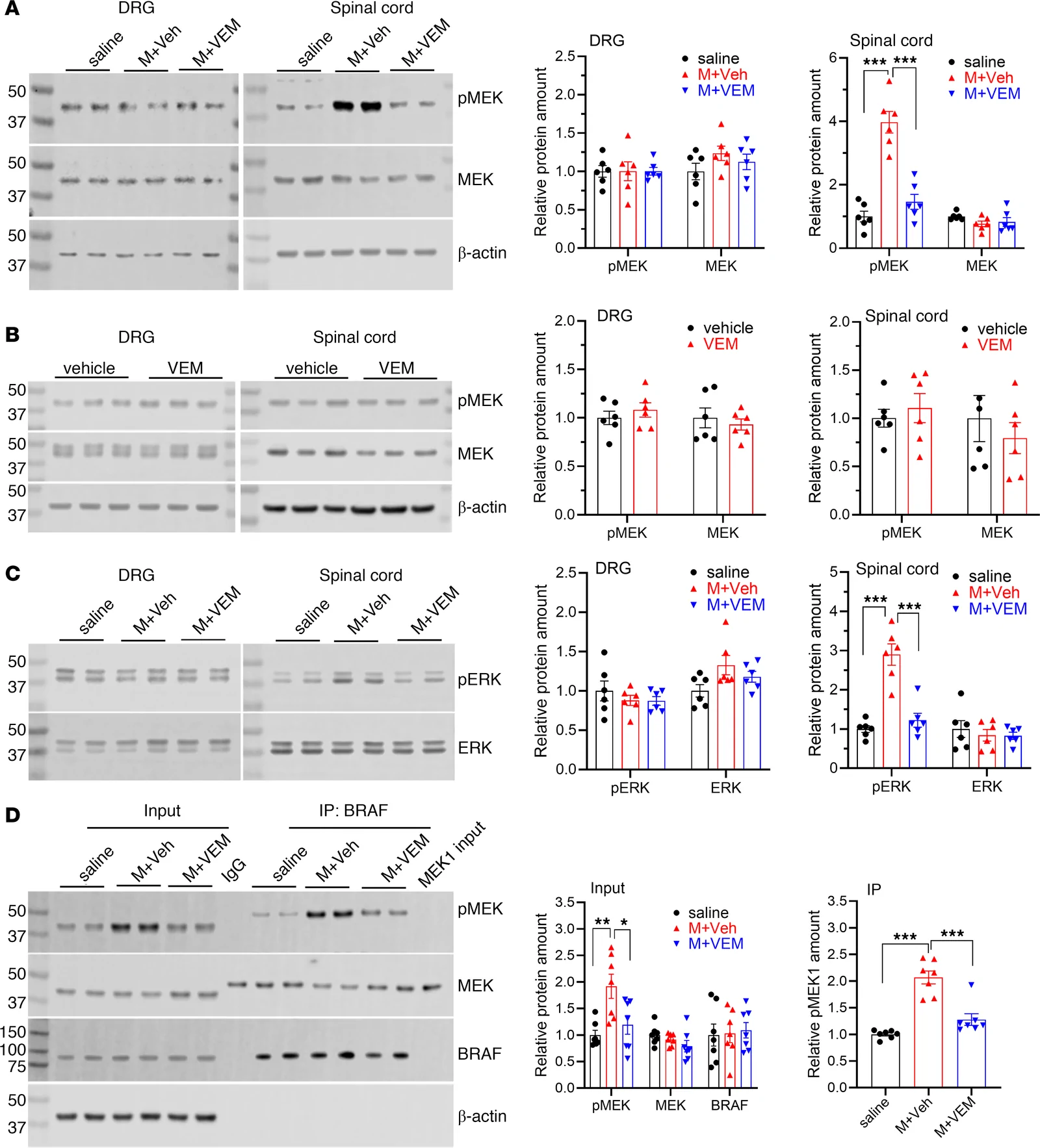
Treatment with morphine enhances BRAF activity in the spinal cord. (**A**) Representative immunoblot images and quantification show the abundance of pMEK and total MEK in the DRG and dorsal spinal cord from rats treated with saline, morphine (M, 5 mg/kg) + vehicle (Veh), or morphine + vemurafenib (VEM, 10 mg/kg) twice daily for 7 days. Total proteins from lumbar DRGs and dorsal spinal cords were extracted for immunoblotting analysis of pMEK and MEK (*n* = 6 rats per group). (**B**) Representative immunoblot images and quantification showing the effect of vemurafenib on pMEK and total MEK expression in the DRG and dorsal spinal cords. Rats were treated with vehicle or vemurafenib (10 mg/kg, i.p.) twice daily for 7 days (*n* = 6 rats per group). (**C**) Representative immunoblot images and quantification show the abundance of pERK and total ERK expression in the DRG and dorsal spinal cords of rats treated with saline, morphine (M) + vehicle (Veh), or morphine + vemurafenib (VEM). Total proteins from lumbar DRGs and dorsal spinal cords were extracted for immunoblotting analysis of pERK and ERK (*n* = 6 rats per group). (**D**) Representative immunoblot images and quantification show the amount of phosphorylated MEK1 (pMEK) in BRAF immunoprecipitates from the spinal cord of rats treated with saline, morphine (M) + vehicle (Veh), or morphine + vemurafenib (VEM). Total proteins extracted from lumbar spinal cords were immunoprecipitated with a BRAF antibody, and the precipitated BRAF was incubated with recombinant MEK1 protein to assess MEK phosphorylation (*n* = 7 rats per group). Data are expressed as means ± SEM. *P < 0.05, **P < 0.01, ***P < 0.001. Statistical significance was determined by 2-tailed Student’s *t* test in **B** and by 1-way ANOVA followed by Tukey’s post hoc test in **A**, **C**, and **D**.

To assess BRAF activity, we measured phosphorylation of MEK, a downstream substrate of BRAF. MEK phosphorylation at Ser217/Ser221 is essential for catalytic activation (39). Vemurafenib is a clinically used selective BRAF inhibitor that suppresses both mutant V600E and WT BRAF, with IC_50_ values of 31 nM and 100 nM, respectively (40). Rats received saline, morphine (5 mg/kg, i.p.) + vehicle, or morphine + vemurafenib (10 mg/kg, i.p.), twice daily for 7 days. In the DRG, the total MEK1/2 and pMEK1/2 amounts were comparable across all groups (Figure 3A). In contrast, morphine treatment increased pMEK1/2, but not total MEK1/2, levels in the spinal cord (Figure 3A). Cotreatment with vemurafenib reversed this morphine-induced increase in pMEK1/2 abundance in the spinal cord (Figure 3A). Vemurafenib alone had no effect on basal MEK1/2 or pMEK1/2 levels in either the DRG or spinal cord tissues (Figure 3B). Similarly, morphine treatment did not change total ERK1/2 or pERK1/2 levels in the DRG but increased pERK1/2 levels in the dorsal spinal cord (Figure 3C). Cotreatment with vemurafenib did not affect total ERK1/2 or pERK1/2 levels in the DRG but reversed the morphine-induced increase in pERK1/2 levels in the spinal cord (Figure 3C).

To directly measure spinal BRAF kinase activity augmented by opioid treatment, we immunoprecipitated BRAF proteins from the dorsal spinal cord of rats treated with saline, morphine + vehicle, or morphine + vemurafenib. The precipitated BRAF protein was then incubated with recombinant polyhistidine-tagged MEK1 protein (45 kDa) in a phosphorylation reaction system to induce MEK1 phosphorylation. MEK1 is a preferred substrate of BRAF and responds rapidly to extracellular stimuli (41). The basal phosphorylation level of recombinant MEK1 (pMEK1) induced by BRAF from saline-treated rats was low (Figure 3D). The pMEK1 level in the spinal cord from the morphine + vehicle group was substantially higher and was normalized by cotreatment with vemurafenib. Notably, the total BRAF and MEK1/2 protein levels in the spinal cord tissues (input) were comparable among the 3 groups (Figure 3D). These findings collectively indicate that opioid treatment increases BRAF activity in the spinal cord and that vemurafenib is effective in inhibiting this opioid-induced neural BRAF overactivity.

### BRAF mediates morphine-induced NMDAR phosphorylation in the spinal cord.

**Figure 4:**
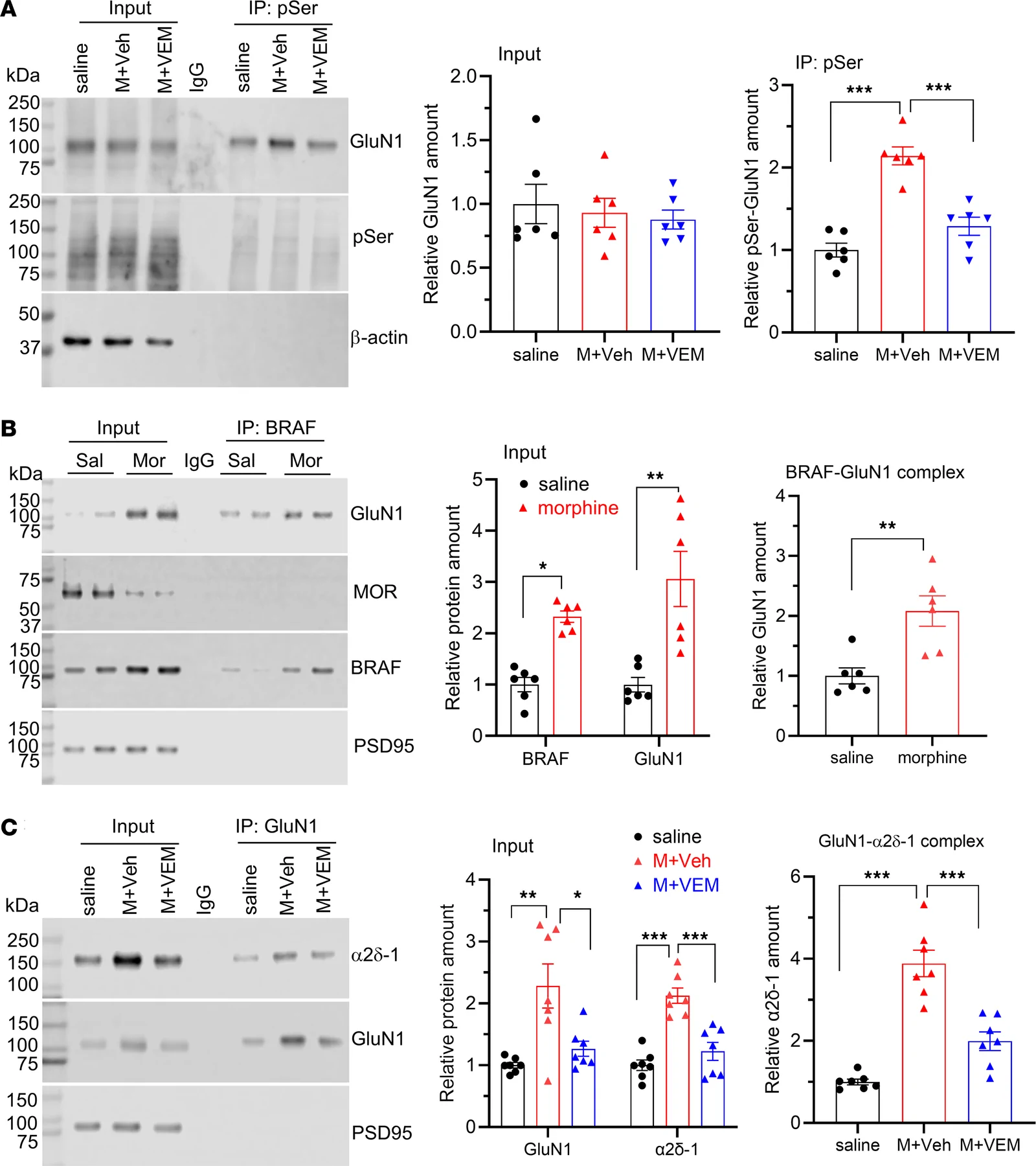
BRAF activity mediates morphine-induced NMDAR phosphorylation and synaptic trafficking of α2δ-1–NMDAR complexes in the spinal cord. (**A**) Representative immunoblot images and quantification of serine-phosphorylated (pSer) GluN1 in the spinal cord (*n* = 6 rats per group) of rats treated with saline, morphine (M) + vehicle (Veh), or morphine + vemurafenib (VEM). (**B**) Representative immunoblot images and quantification show that BRAF interacted with GluN1, but not μ-opioid receptor (MOR), in the synaptosomal fraction of the spinal cord (*n* = 6 rats per group) from rats treated with saline (Sal) or morphine (Mor). (**C**) Representative immunoblot images and quantification show the protein amount of α2δ-1, GluN1, and α2δ-1–GluN1 complexes in the spinal cord synaptosome from rats (*n* = 6 rats per group) treated with saline, morphine (M) + vehicle (Veh), or morphine + vemurafenib (VEM). Total and synaptosomal proteins were extracted from the lumbar dorsal spinal cord for immunoprecipitation with pSer, BRAF, or GluN1 antibodies. The immunoprecipitated proteins and input proteins were used for immunoblotting analysis. β-actin and PSD95 were used as the loading control. Data are expressed as means ± SEM. *P < 0.05, **P < 0.01, ***P < 0.001. Statistical significance was determined by 2-tailed Student’s *t* test for **B** and by 1-way ANOVA followed by Tukey’s post hoc test for **A** and **C**.

Increased NMDAR phosphorylation in the spinal cord plays a key role in opioid-induced tolerance and hyperalgesia (19, 20). We next determined whether BRAF activity is involved in opioid-induced NMDAR phosphorylation in the spinal cord. To this end, lumbar dorsal spinal cords were collected from rats treated for 7 days with saline, morphine + vehicle, or morphine + vemurafenib. Total proteins were extracted and subjected to immunoprecipitation with a phosphoserine (pSer) antibody, followed by immunoblotting for GluN1, an obligatory NMDAR subunit (42). A single GluN1 protein band was detected in the immunoprecipitates by the pSer antibody, but not by irrelevant IgG control. Compared with saline control, morphine treatment markedly increased GluN1 levels in the pSer precipitates (Figure 4A), indicating enhanced GluN1 serine phosphorylation. Furthermore, cotreatment with vemurafenib strongly inhibited this morphine-induced increase. In contrast, the total GluN1 levels in input lysates did not differ among the 3 groups (Figure 4A). These results suggest that BRAF activity is involved in morphine-induced NMDAR phosphorylation in the spinal cord.

### Morphine treatment increases synaptic trafficking of BRAF-NMDAR complexes in the spinal cord.

Treatment with morphine enhances the physical association of ERK1/2 with NMDARs in the spinal cord (18). We used coimmunoprecipitation to determine whether BRAF is physically associated with NMDARs and MORs in the spinal cord from rats treated with saline or morphine for 7 days. Synaptosomal proteins extracted from rat spinal cord tissues were immunoprecipitated with a BRAF antibody followed by immunoblotting for GluN1. GluN1 was detected in the precipitates obtained using a BRAF antibody (Figure 4B). The abundance of BRAF-GluN1 protein complexes, as well as the total protein amount of BRAF and GluN1 in spinal cord synaptosomes (the input), were substantially higher in morphine-treated rats than in saline-treated rats (Figure 4B). However, GluN1 was not present in the BRAF immunoprecipitates in the DRG tissue from rats treated with saline or morphine (Supplemental Figure 2). Furthermore, MOR was not detected in BRAF immunoprecipitates from either the spinal cord (Figure 4B) or DRG (Supplemental Figure 2) of rats treated with saline or morphine, indicating that BRAF does not directly interact with MORs in these tissues. These data suggest that opioid treatment promotes BRAF-NMDAR interactions and their trafficking at spinal cord synapses.

Additionally, coimmunoprecipitation analysis revealed that the GluN1 antibody, but not the control IgG, precipitated both BRAF and ERK1/2 from lumbar spinal cord tissues of 2 human donors (Supplemental Figure 3). Notably, the amount of BRAF protein coprecipitated by GluN1 appeared to be greater in the donor who died of methadone intoxication than in the donor who died of generalized dystonia (Supplemental Figure 3), suggesting that opioids may also enhance the association between BRAF and NMDARs in the human spinal cord.

### Morphine treatment promotes synaptic trafficking of α2δ-1–bound NMDARs in the spinal cord through BRAF.

α2δ-1 is a recently identified interacting protein of NMDARs and AMPARs (43–45). It preferentially binds to phosphorylated NMDARs, facilitating their synaptic trafficking in conditions such as neuropathic pain and opioid-induced hyperalgesia (16, 45–48). To determine whether BRAF activity contributes to morphine-induced synaptic expression of α2δ-1–bound NMDARs, we analyzed synaptosomal proteins from the dorsal spinal cord of rats treated for 7 days with saline, morphine + vehicle, or morphine + vemurafenib. Immunoblots revealed that morphine treatment increased GluN1 and α2δ-1 levels in spinal cord synaptosomes compared with saline controls, and these increases were largely normalized by vemurafenib cotreatment (Figure 4C). Furthermore, in the GluN1 immunoprecipitates, morphine treatment substantially elevated α2δ-1 levels, indicating enhanced α2δ-1–NMDAR interactions. Cotreatment with vemurafenib largely diminished this morphine-induced increase in α2δ-1–NMDAR complexes (Figure 4C). These findings suggest that BRAF activity plays a critical role in opioid-induced synaptic trafficking of α2δ-1–bound NMDARs in the spinal cord.

### BRAF mediates morphine-induced hyperactivity of NMDARs at primary afferent terminals in the spinal cord.

**Figure 5:**
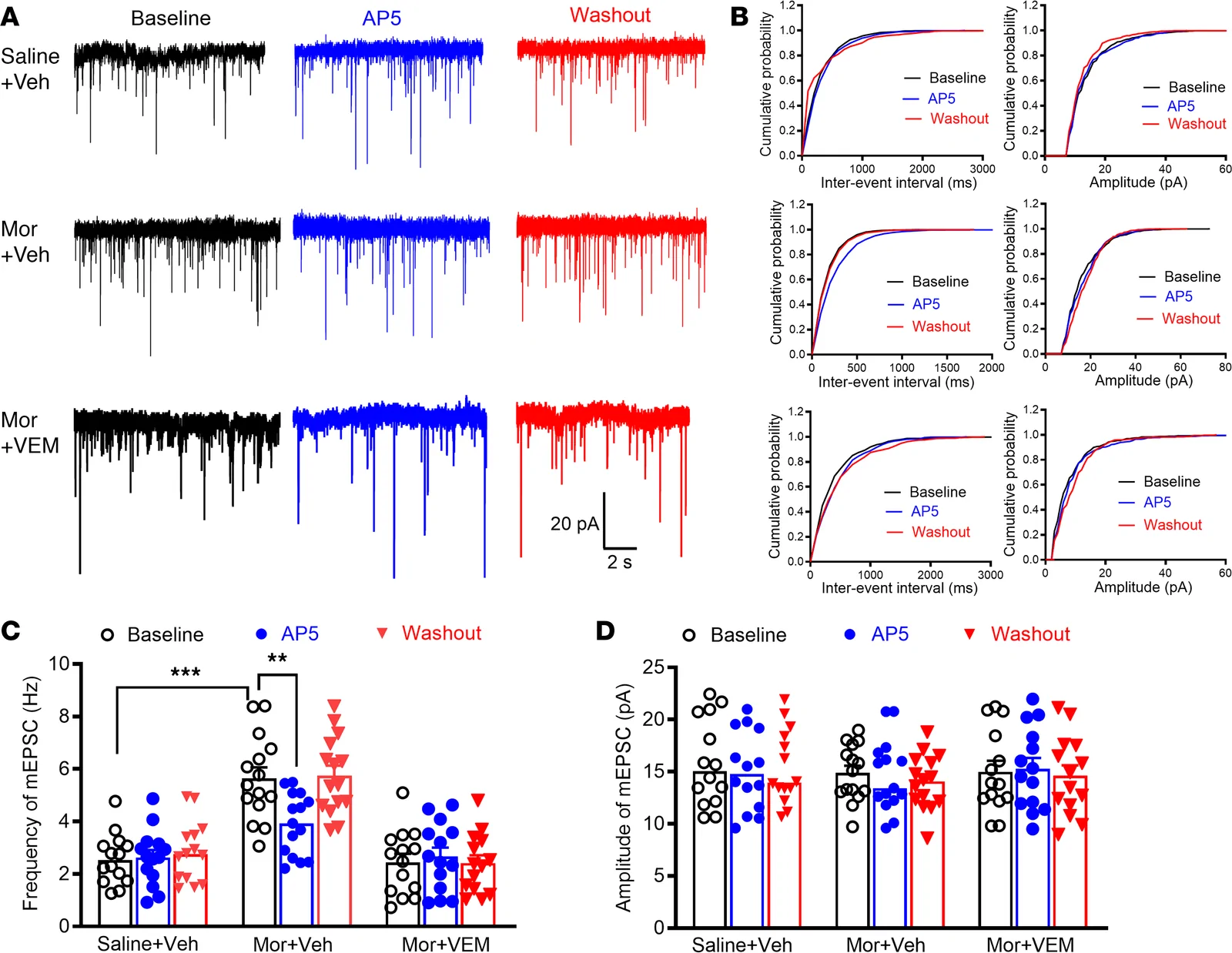
Morphine treatment increases NMDAR-dependent frequency of mEPSCs in spinal dorsal horn neurons through BRAF. (**A**–**D**) Representative recording traces (**A**), cumulative plots (**B**), and summary data (**C** and **D**) show the effect of bath application of 50 μM AP5 on the frequency and amplitude of mEPSCs in lamina II neurons from rats treated with saline or morphine (5 mg/kg) twice daily for 7 days. Spinal cord slices were treated with vemurafenib (VEM, 5 μM) or vehicle (Veh) for 30 minutes immediately before recording. Recordings of mEPSCs were obtained from 5 rats per group (*n* = 14 neurons for saline + Veh group, *n* = 15 neurons for Mor + Veh group, *n* = 14 neurons for Mor + VEM group). Data are shown as means ± SEM. **P < 0.01, ***P < 0.001 (2-way ANOVA followed by Tukey’s post hoc test).

Hyperactivity of presynaptic NMDARs in the spinal dorsal horn is a critical mechanism underlying opioid-induced hyperalgesia and tolerance (3, 12, 15). To assess whether BRAF activity contributes to this process, we performed electrophysiological recordings in lamina II neurons from spinal cord slices of rats treated with saline or morphine for 7 days. The baseline frequency — but not amplitude — of miniature excitatory postsynaptic currents (mEPSCs) was notably higher in morphine-treated rats than in saline controls (Figure 5). Bath application of 50 μM 2-amino-5-phosphonopentanoic acid (AP5), a specific NMDAR antagonist, for 6 minutes rapidly decreased the frequency of mEPSCs in spinal cord slices from morphine-treated rats but had no such effect on saline-treated rats. Treatment of spinal cord slices with 5 μM vemurafenib for 30 minutes reversed the elevated baseline frequency of mEPSCs from morphine-treated rats (Figure 5). In these spinal cord slices treated with vemurafenib, AP5 application had no further effect on the mEPSCs.

**Figure 6:**
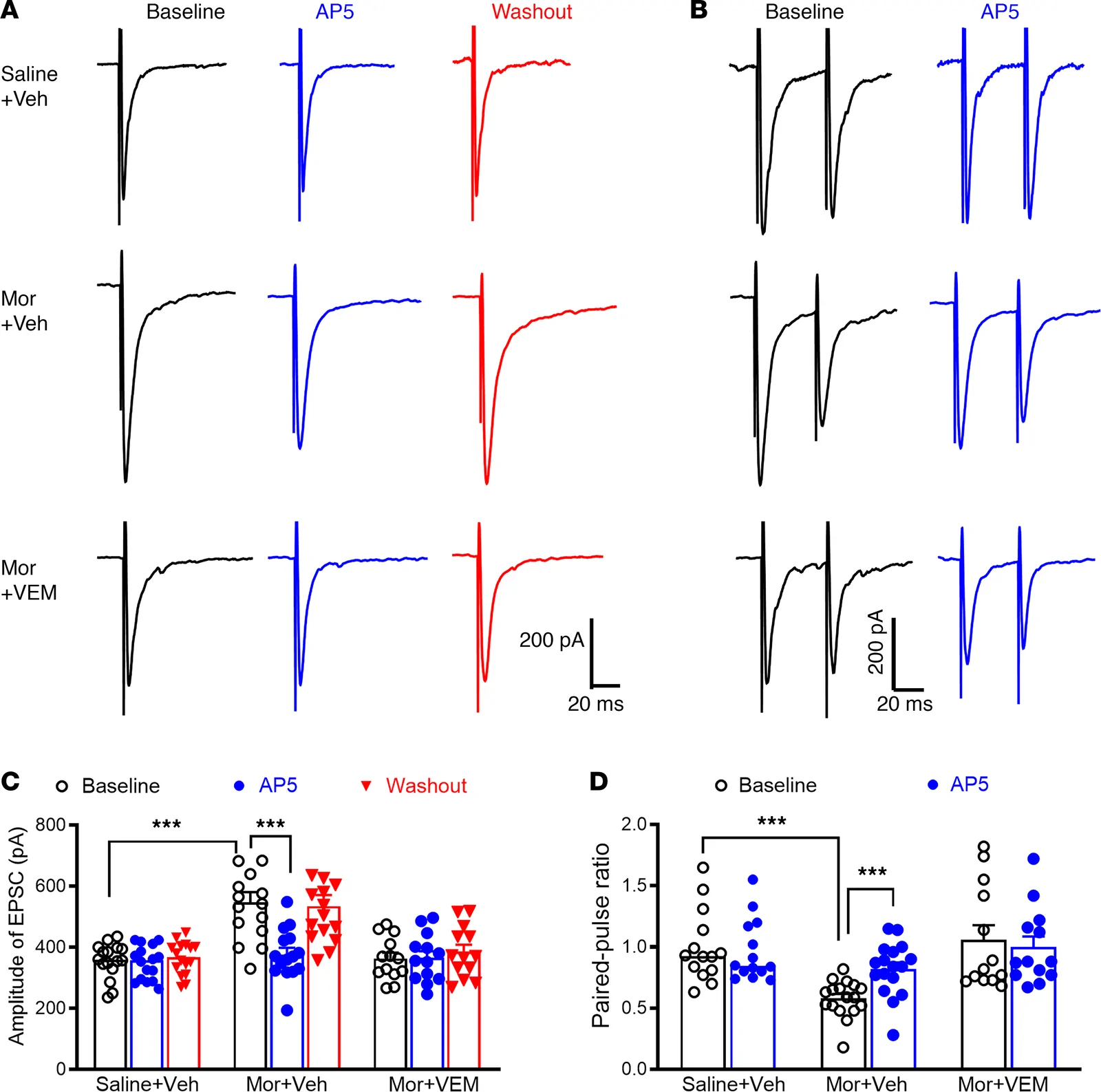
Morphine treatment enhances the amplitude of dorsal root–evoked EPSCs and paired-pulse ratio of EPSCs in the spinal cord through BRAF. (**A**–**D**) Representative recording traces (**A** and **B**) and summary data (**C** and **D**) show the effect of bath application of 50 μM AP5 on the amplitude of dorsal root–evoked EPSCs and paired-pulse ratio (PPR) in lamina II neurons from rats treated with saline or morphine (5 mg/kg) twice daily for 7 days. Spinal cord slices were treated with vemurafenib (VEM, 5 μM) or vehicle (Veh) for 30 minutes immediately before recording. Monosynaptic EPSCs were elicited by electrical stimulation of the dorsal root. Recordings of evoked EPSCs (*n* = 16 neurons for saline + Veh group, *n* = 15 neurons for Mor + Veh group, *n* = 13 neurons for Mor + VEM group) and the PPR (*n* = 14 neurons for saline + Veh group, *n* = 17 neurons for Mor + Veh group, *n* = 13 neurons for Mor + VEM) were obtained from 5 rats per group. Data are shown as mean ± SEM. ***P < 0.001 (2-way ANOVA followed by Tukey’s post hoc test).

We next determined the effect of vemurafenib on EPSCs in spinal lamina II neurons monosynaptically evoked from the dorsal root stimulation. The baseline amplitude of evoked EPSCs was greater in morphine-treated rats than in saline-treated rats. Bath application of AP5 readily decreased the amplitude of evoked EPSCs in morphine-treated rats but had no effect in saline-treated rats (Figure 6). Treatment of spinal cord slices with vemurafenib normalized the elevated baseline amplitude of evoked EPSCs in morphine-treated rats to a level similar to that in saline-treated rats (Figure 6). In spinal cord slices pretreated with vemurafenib, subsequent application of AP5 had no further effect on the amplitude of evoked EPSCs.

We also examined the paired-pulse ratio (PPR) of EPSCs in spinal lamina II neurons monosynaptically evoked from dorsal root stimulation, which reflects the probability of glutamate release from presynaptic terminals based on responses to paired stimuli (15, 16). The baseline PPR was notably lower in morphine-treated rats than in saline-treated rats. Bath application of AP5 preferentially reduced the first evoked EPSCs more than the second, resulting in an increased PPR in morphine-treated rats (Figure 6). However, AP5 had no effect on the PPR in saline-treated rats. Pretreatment with vemurafenib restored the reduced baseline PPR in morphine-treated rats (Figure 6), and subsequent AP5 application produced no additional effect. Together, these results suggest that increased BRAF activity mediates opioid-induced presynaptic NMDAR hyperactivity at primary afferent terminals in the spinal dorsal horn.

### Inhibition of BRAF or MEK reduces morphine-induced hyperalgesia and tolerance.

**Figure 7:**
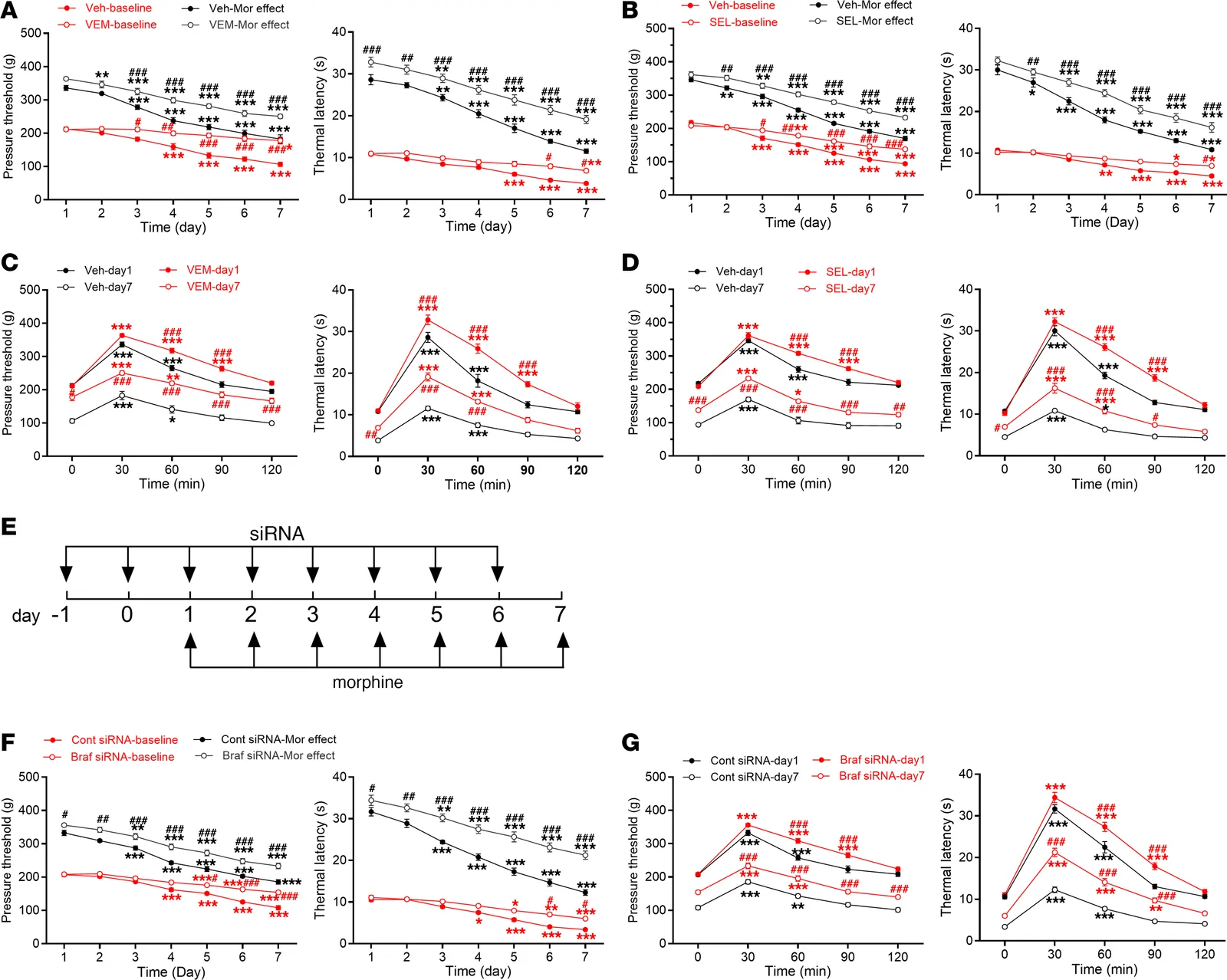
Inhibition of BRAF or MEK activity or BRAF knockdown reduces morphine-induced hyperalgesia and tolerance. (**A** and **B**) Time course of the effect of vemurafenib (VEM, **A**) or selumetinib (SEL, **B**) on morphine-induced hyperalgesia and tolerance. Rats were intraperitoneally injected with morphine (Mor, 5 mg/kg) twice daily for 7 days. VEM (10 mg/kg) or SEL (10 mg/kg) was intraperitoneally injected 15 minutes prior to each morphine injection daily. Nociceptive threshold and latency were tested before (baseline) and 30 minutes after the first morphine injection daily. (**C** and **D**) Time course of the acute analgesic effect of morphine in rats cotreated with VEM (**C**) or SEL (**D**). Nociceptive threshold and latency were tested after the first morphine injection on day 1 and day 7. (**E**) Flowchart diagram shows the timeline of injection of siRNA (3 μg, intrathecally, once daily) and morphine (5 mg/kg, i.p., twice daily) used for behavioral testing. *Braf*-specific siRNA or control siRNA were injected 15 minutes prior to the first morphine injection daily. (**F**) Time course of morphine-induced hyperalgesia and tolerance in rats treated with *Braf*-specific siRNA or control (Cont) siRNA (*n* = 9 rats per group). Pressure withdrawal threshold and thermal withdrawal latency were measured before (baseline) and 30 minutes after the first morphine (Mor) injection daily on days 1–7. (**G**) Time course of the acute analgesic effect produced by the first morphine injection on day 1 and day 7 in rats treated with *Braf*-specific siRNA or control (Cont) siRNA (*n* = 9 rats per group). *P < 0.05, **P < 0.01, ***P < 0.001 versus respective values at day 1 or time (min) 0; ^#^*P* < 0.05, ^##^*P* < 0.01, ^###^*P* < 0.001 versus respective control groups at the same time point (2-way ANOVA followed by Tukey’s post hoc test).

To determine whether BRAF activity contributes to morphine-induced hyperalgesia and tolerance, adult rats were treated with morphine (5 mg/kg, i.p., twice daily) for 7 consecutive days. Vemurafenib (10 mg/kg, i.p.) or vehicle was administered 15 minutes prior to each morphine injection. Withdrawal threshold and latency in response to noxious pressure and radiant heat stimuli were measured 30 minutes before (baseline) and 30 minutes after the first daily morphine injection. Daily morphine administration in vehicle-treated rats produced a gradual reduction in baseline withdrawal threshold and latency, indicating mechanical and thermal hyperalgesia (Figure 7A). These rats also exhibited a progressive decline in the analgesic effect of morphine, reflecting the development of analgesic tolerance (Figure 7A). Cotreatment with vemurafenib substantially attenuated both the reduction in baseline withdrawal threshold and latency and the loss in morphine analgesic efficacy (Figure 7A).

To determine whether MEK activity, which is downstream of BRAF, also mediates morphine-induced hyperalgesia and tolerance, we administered selumetinib (10 mg/kg, i.p.), a clinically available MEK1/2 inhibitor (49), 15 minutes prior to morphine (5 mg/kg, i.p.) twice daily for 7 days. Similar to vemurafenib, selumetinib markedly attenuated reductions in baseline withdrawal threshold and latency and preserved morphine’s analgesic effect (Figure 7B).

Furthermore, we examined the time course of the acute analgesic response to morphine on the first and seventh treatment days. In the vehicle group, morphine (5 mg/kg, i.p.) produced a transient analgesic effect on both days, reflected by increased pressure withdrawal threshold and thermal withdrawal latency, lasting for 90–120 minutes after injection (Figure 7C). Notably, cotreatment with vemurafenib potentiated these morphine-induced increases in withdrawal threshold and latency on both days (Figure 7C). Similarly, cotreatment with selumetinib potentiated morphine-induced increases on both days (Figure 7D). These findings suggest that the increased BRAF activity, along with its downstream MEK signaling, counteracts opioid analgesia and contributes to the development of opioid-induced hyperalgesia and tolerance.

### siRNA-mediated BRAF knockdown at the spinal cord level attenuates morphine-induced hyperalgesia and tolerance.

To determine whether spinal BRAF contributes to opioid-induced hyperalgesia and tolerance, we intrathecally injected 3 μg of *Braf*-specific siRNA or control siRNA in rats once daily for 7 days and then collected the lumbar DRG and dorsal spinal cord tissue to confirm the knockdown efficacy. Intrathecally injected siRNA knocks down the target in both the DRG and spinal cord tissues (50, 51). As expected, compared with the control siRNA, *Braf*-specific siRNA markedly decreased BRAF protein levels in both the DRG and spinal cord tissues (Supplemental Figure 4).

Next, we intrathecally injected *Braf*-specific siRNA or control siRNA in rats for 2 days followed by concurrent treatment with morphine (5 mg/kg, i.p., twice daily) for 7 days (Figure 7E). Compared with control siRNA, *Braf*-specific siRNA mitigated morphine-induced reductions in baseline pressure withdrawal threshold and thermal withdrawal latency and attenuated the decline in the analgesic effect of morphine (Figure 7F). Furthermore, *Braf*-specific siRNA potentiated morphine-induced increases in withdrawal threshold and latency on both days 1 and 7 (Figure 7G). These findings suggest that BRAF at the spinal cord level mediates opioid-induced hyperalgesia and tolerance and opposes opioid analgesia.

### Conditional Braf knockout in DRG neurons attenuates morphine-induced hyperalgesia and tolerance.

**Figure 8:**
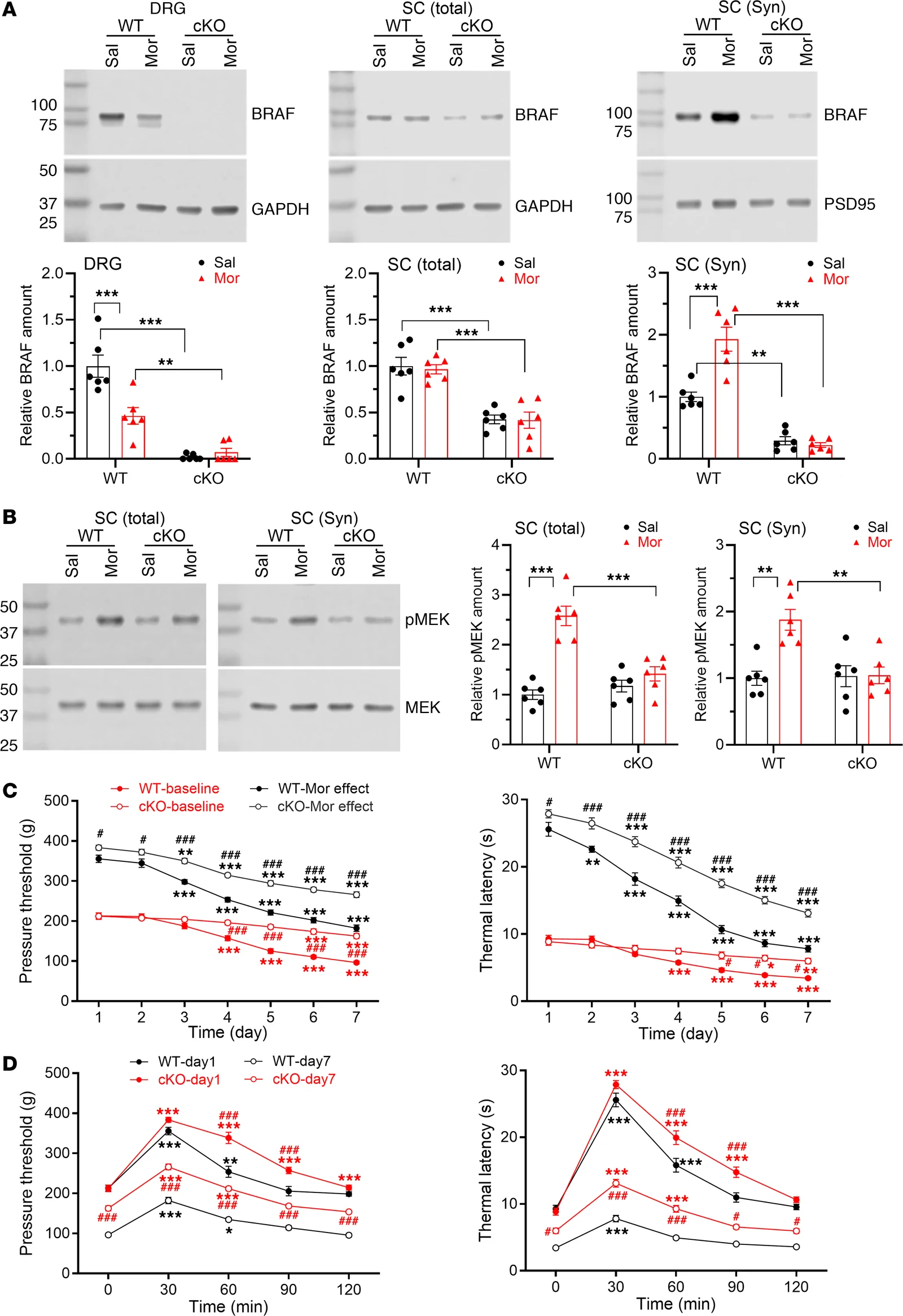
*Braf* knockout in primary sensory neurons attenuates morphine-induced BRAF translocation as well as hyperalgesia and tolerance. (**A**) Representative immunoblot images and quantification show BRAF protein expression in the DRG and spinal cord (SC) of conditional *Braf* knockout (cKO) and WT mice treated with saline (Sal) or morphine (Mor, 10 mg/kg, twice daily) for 7 days (*n* = 6 mice per group). Total and synaptosomal (Syn) proteins were extracted from lumbar DRG and dorsal spinal cord tissues for immunoblotting. GAPDH or PSD95 was used as a loading control. (**B**) Representative immunoblot images and quantification show the amount of pMEK and total MEK in the spinal cord from WT and *Braf*-cKO mice treated with saline or morphine (10 mg/kg, i.p., twice daily) for 7 consecutive days (*n* = 6 mice per group). Total and synaptosomal (Syn) proteins from the dorsal spinal cord were extracted for immunoblotting. (**C**) Time course of hyperalgesia and tolerance in WT (*n* = 11 mice) and *Braf*-cKO (*n* = 13 mice) treated with morphine (10 mg/kg, twice daily) for 7 days. Pressure withdrawal threshold and heat withdrawal latency were measured before (baseline) and 30 minutes after the first morphine (Mor) injection daily. (**D**) Time course of the acute analgesic effect produced by the first morphine injection on day 1 and day 7 in WT (*n* = 11 mice) and *Braf*-cKO (*n* = 13 mice). Data are expressed as means± SEM. In Panels **A** and **B**, **P < 0.01, ***P < 0.001 (1-way ANOVA followed by Tukey’s post hoc test). In **C** and **D**, *P < 0.05, **P < 0.01, ***P < 0.001 versus respective values at day 1 or time (min) 0. ^#^*P* < 0.05, ^###^*P* < 0.001 versus respective WT groups at the same time point (2-way ANOVA followed by Tukey’s post hoc test).

MORs and NMDARs expressed in DRG neurons play critical roles in opioid-induced hyperalgesia and tolerance (11, 12). We therefore investigated whether BRAF in DRG neurons is required for these processes. To this end, we crossed *Braf*^Fl+/+^ (52) and *Avil*^iCre/ERT2+^ (53) mice to generate *Braf*^Fl+/+^:*Avil*^iCre/ERT2+^ mice and administered tamoxifen to induce conditional *Braf* knockout (*Braf*-cKO) in DRG neurons. As expected, *Braf-*cKO mice exhibited near complete loss of BRAF expression in the DRG (Figure 8A). BRAF protein levels in spinal cord tissues and spinal cord synaptosomal fractions were also markedly lower in *Braf-*cKO mice than in WT mice. In WT mice, morphine treatment increased BRAF expression in spinal cord synaptosomes. However, morphine failed to increase BRAF levels in spinal cord synaptosomes in *Braf-*cKO mice (Figure 8A).

We next determined whether BRAF in DRG neurons contributes to morphine-induced BRAF overactivity in the spinal cord. *Braf*-cKO and WT mice were treated with saline or morphine (10 mg/kg, i.p., twice daily for 7 days), and total and synaptosomal proteins were extracted from lumbar dorsal spinal cords. The total and synaptosomal MEK1/2 protein levels were similar between WT and *Braf*-cKO mice treated with saline. In WT mice, morphine treatment increased pMEK1/2 levels in both total lysates and synaptosomal fractions (Figure 8B), consistent with data from rats. This morphine-induced increase was diminished in *Braf*-cKO mice (Figure 8B), indicating that BRAF from DRG neurons drives the morphine-induced BRAF overactivity in the spinal cord.

We then determined whether BRAF in DRG neurons mediates morphine-induced hyperalgesia and tolerance. *Braf*-cKO and WT mice were treated with morphine (10 mg/kg, twice daily) for 7 days, and hindpaw withdrawal threshold and latency were measured before (baseline) and 30 minutes after the first daily morphine injection. Acute analgesic effect of the first morphine injection was also assessed on days 1 and 7. Baseline mechanical and thermal thresholds were comparable between *Braf*-cKO and WT mice prior to morphine treatment (Figure 8C). In WT mice, morphine treatment produced progressive reductions in baseline withdrawal thresholds and analgesic efficacy, indicative of hyperalgesia and tolerance. These reductions were attenuated in *Braf*-cKO mice (Figure 8C).

Furthermore, morphine injection transiently increased pressure withdrawal threshold and heat withdrawal latency on both days 1 and 7 in both genotypes (Figure 8D). Notably, acute analgesic effects of morphine were greater in *Braf*-cKO mice than in WT mice on both days (Figure 8D). These results suggest that BRAF originating from DRG neurons promotes opioid-induced hyperalgesia and tolerance and antagonizes opioid analgesia.

### BRAF from DRG neurons mediates morphine-induced NMDAR phosphorylation and synaptic trafficking of α2δ-1–bound NMDARs in the spinal cord.

**Figure 9:**
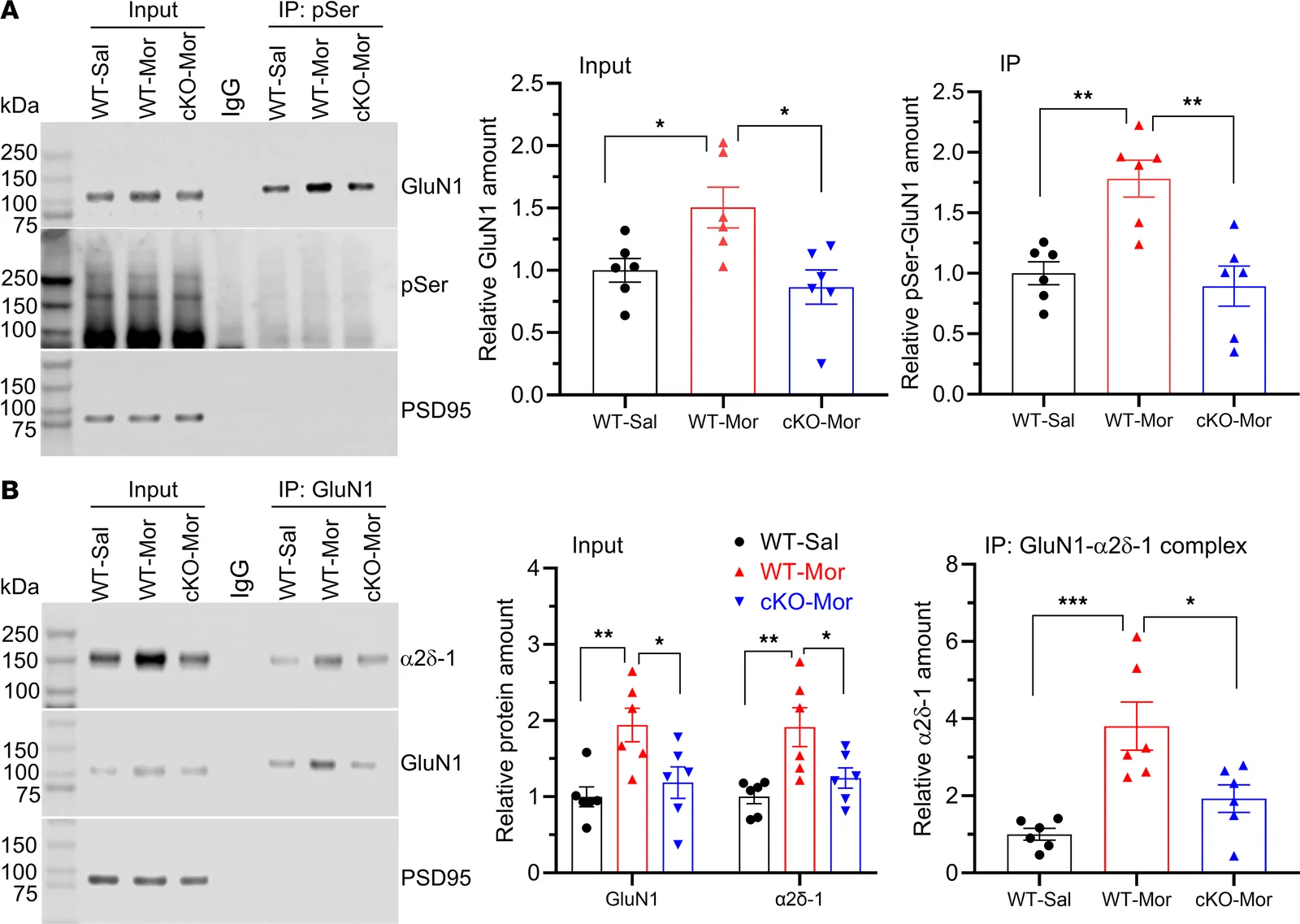
*Braf* knockout in primary sensory neurons blocks morphine-induced NMDAR phosphorylation and synaptic trafficking of α2δ-1–bound NMDARs in the spinal cord. (**A)** Representative immunoblot images and quantification show the amount of total GluN1 and phosphorylated GluN1 from spinal cord synaptosomes of WT mice treated with saline (Sal) or morphine (Mor) as well as *Braf*-cKO mice treated with morphine (*n* = 6 mice per group). (**B**) Representative immunoblot images and quantification show the amount of α2δ-1, GluN1, and α2δ-1–GluN1 complexes in spinal cord synaptosomes from WT mice treated with saline (Sal) or morphine (Mor) as well as *Braf*-cKO mice treated with morphine (*n* = 6 mice per group). Synaptosomal proteins from the dorsal spinal cord were immunoprecipitated (IP) with a pSer or GluN1 antibody. Both input and precipitated proteins were then immunoblotted for α2δ-1 and GluN1. PSD95 was used as a loading control. Data are presented as means ± SEM. *P < 0.05, **P < 0.01, ***P < 0.001 (1-way ANOVA followed by Tukey’s post hoc test).

We next investigated whether BRAF from DRG neurons contributes to morphine-induced NMDAR phosphorylation and synaptic expression in the spinal cord. *Braf*-cKO and WT mice were treated with morphine (10 mg/kg, twice daily) for 7 days. Synaptosomal proteins were extracted from lumbar dorsal spinal cords and subjected to immunoprecipitation using a pSer antibody. Both total synaptosomal proteins (input) and pSer-precipitated proteins were analyzed by immunoblotting with a GluN1 antibody. Morphine treatment increased GluN1 levels in both input and pSer precipitates in WT mice compared with saline-treated WT mice (Figure 9A). In contrast, morphine treatment failed to increase GluN1 levels in either input or pSer precipitates in *Braf*-cKO mice (Figure 9A). These data suggest that BRAF expressed in DRG neurons is essential for opioid-induced enhancement of NMDAR phosphorylation and synaptic trafficking in the spinal cord.

Given that *Braf*-cKO in DRG neurons attenuated morphine-induced NMDAR phosphorylation and synaptic trafficking in the spinal cord, we investigated whether DRG neuron–derived BRAF contributes to morphine-induced synaptic expression of α2δ-1–bound NMDARs in the spinal cord. Synaptosomal proteins were extracted from dorsal spinal cord tissues of *Braf*-cKO and WT mice treated with morphine for 7 days. Total synaptosomal proteins (input) and GluN1-immunoprecipitated proteins were analyzed by immunoblotting with GluN1 and α2δ-1 antibodies. Consistent with rat data, morphine markedly increased total α2δ-1 levels in synaptosomal input from WT mice. In WT mice, morphine also substantially increased α2δ-1 levels in GluN1 immunoprecipitates (Figure 9B). Remarkably, morphine-induced increases in α2δ-1 amount and α2δ-1–GluN1 complexes in spinal cord synaptosomes were diminished in *Braf*-cKO mice (Figure 9B). These findings suggest that opioids potentiate synaptic expression of α2δ-1–bound NMDARs in the spinal cord through BRAF signaling in DRG neurons.

### Conditional Braf knockout in DRG neurons diminishes morphine-induced presynaptic NMDAR hyperactivity in the spinal dorsal horn.

**Figure 10:**
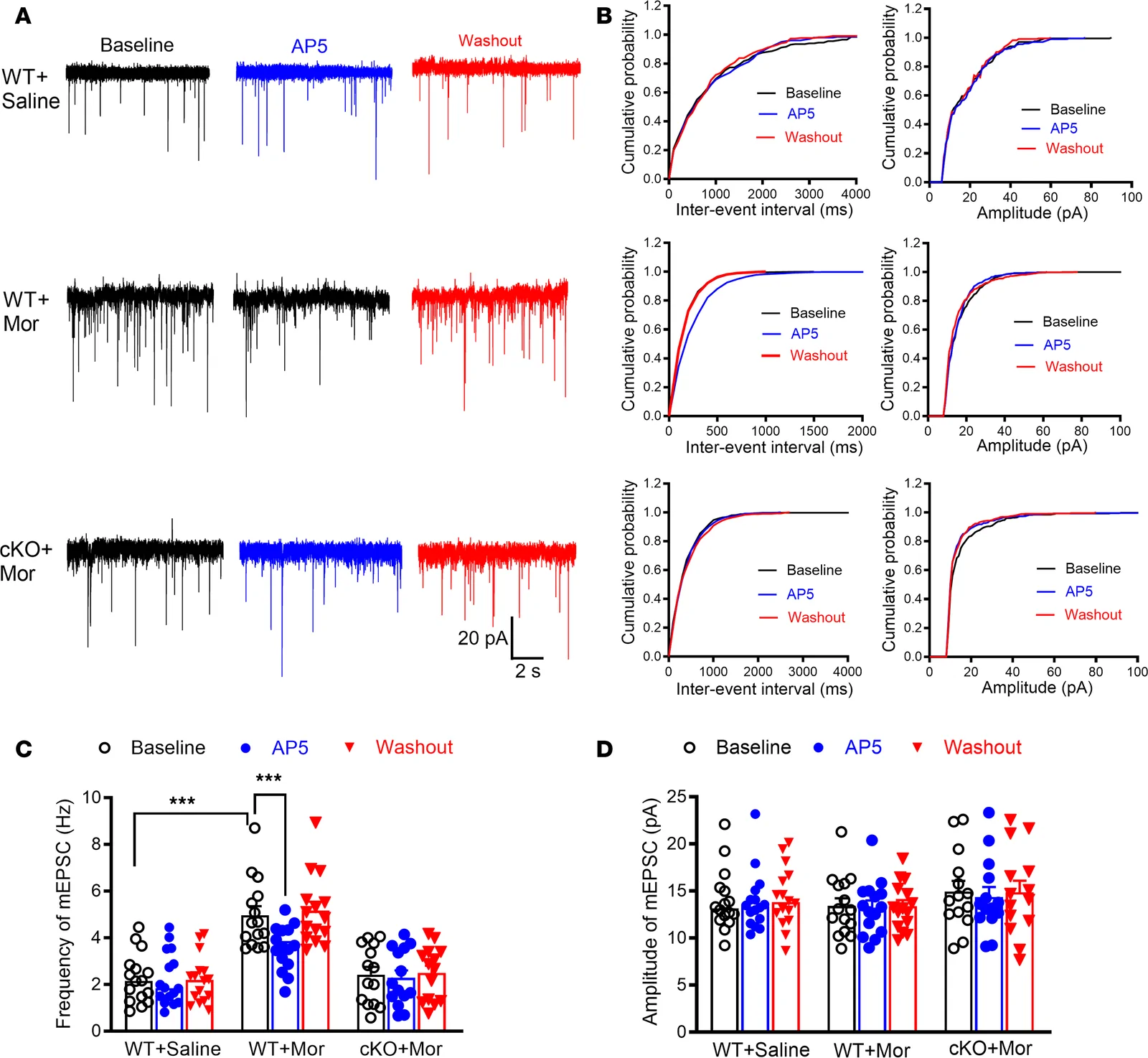
BRAF in primary sensory neurons underlies the morphine-induced increase in the frequency of NMDAR-dependent mEPSCs in spinal dorsal horn neurons. (**A**–**D**) Representative recording traces (**A**), cumulative plots (**B**), and summary data (**C** and **D**) show the effect of bath application of 50 μM AP5 on the frequency and amplitude of mEPSCs in lamina II neurons from WT and conditional *Braf* knockout (cKO) mice treated with saline or morphine (Mor, 10 mg/kg twice daily) for 7 days. Recordings of mEPSCs were obtained from 5 mice per group (*n* = 16 neurons for WT + saline group, *n* = 15 neurons for WT + Mor group, *n* = 14 neurons for cKO + Mor group). Data presented are means ± SEM. ***P < 0.001, 2-way ANOVA followed by Tukey’s post hoc test.

Finally, we determined the role of BRAF from DRG neurons in morphine-induced hyperactivity of presynaptic NMDARs in spinal dorsal horn neurons. *Braf*-cKO and WT mice were treated with morphine for 7 days, and lumbar spinal cord slices were used for electrophysiological recordings. The baseline frequency, but not amplitude, of mEPSCs in lamina II neurons was substantially higher in morphine-treated WT mice than in saline-treated WT mice (Figure 10). The morphine-induced increase in mEPSCs frequency in WT mice was rapidly reduced by bath application of AP5. In *Braf*-cKO mice, however, morphine treatment did not increase baseline mEPSC frequency, and subsequent AP5 application had no effect (Figure 10).

**Figure 11:**
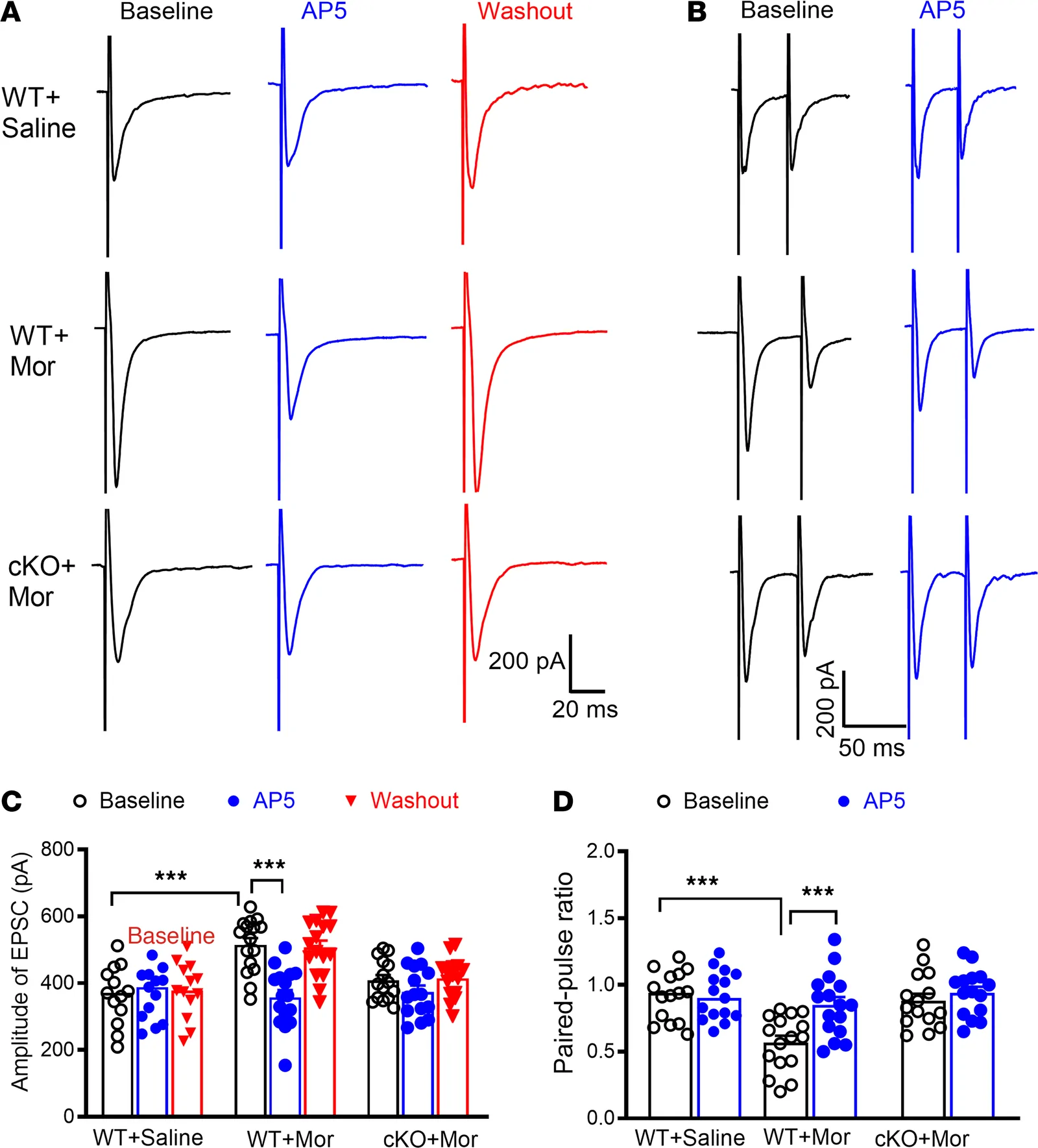
*Braf* knockout in primary sensory neurons eliminates morphine-induced changes in the amplitude of dorsal root–evoked EPSCs and paired-pulse ratio of EPSCs in spinal dorsal horn neurons. (**A**–**D**) Representative current traces (**A** and **B**) and quantification (**C** and **D**) show the effect of bath application of 50 μM AP5 on the amplitude of dorsal root–evoked EPSCs and paired-pulse ratio (PPR) of EPSCs in lamina II neurons from WT and conditional *Braf* knockout (cKO) mice treated with saline or morphine (Mor, 10 mg/kg twice daily) for 7 days. Recordings of evoked EPSCs (*n* = 13 neurons for WT + saline group, *n* = 16 neurons for WT + morphine group, *n* = 15 neurons for cKO + morphine group) and the PPR (*n* = 15 neurons for WT + saline group, *n* = 16 neurons for WT + morphine group, *n* = 15 neurons for cKO + morphine group) were obtained from 5 mice per group. Data presented are means ± SEM. ***P < 0.001; 2-way ANOVA followed by Tukey’s post hoc test.

In WT mice, morphine treatment also increased the baseline amplitude of EPSCs in lamina II neurons monosynaptically evoked by dorsal root stimulation compared with saline-treated WT mice (Figure 11). In contrast, morphine treatment did not affect the baseline amplitude of evoked EPSCs in lamina II neurons of *Braf*-cKO mice. Bath application of AP5 rapidly decreased the amplitude of evoked EPSCs in morphine-treated WT mice but had no effect in saline-treated WT mice or morphine-treated *Braf*-cKO mice (Figure 11).

In addition, morphine treatment decreased baseline PPR of evoked EPSCs in WT mice compared with saline-treated WT mice. Bath application of AP5 increased the PPR of evoked EPSCs in morphine-treated WT mice but had no such effect in saline-treated WT mice (Figure 11). In *Braf-*cKO mice, morphine treatment did not alter baseline PPR, which remained comparable with saline-treated WT mice (Figure 11). Subsequent AP5 application had no observable effect on baseline PPR in *Braf-*cKO mice. Collectively, these results suggest that BRAF from DRG neurons is required for opioid-induced hyperactivity of NMDARs at primary afferent terminals in the spinal dorsal horn.

## Discussion

A major finding of our study is that opioid treatment activates the MEK/ERK signaling pathway by enhancing BRAF activity in the spinal cord. As a core component of the RAS/RAF/MEK/ERK cascade, RAF primarily functions by phosphorylating and activating MEK (54), which, in turn, activates ERK. Activated ERK subsequently phosphorylates a wide range of downstream targets involved in diverse cellular processes. In CHO, COS-7, and HEK293 cell lines transfected with MORs, opioids activate ERK1/2 through Gαi/o-coupled G-proteins and their Gβγ subunits (21–26). ERK inhibition attenuates opioid-induced hyperalgesia and tolerance (18, 55). In this study, we demonstrated that morphine administration markedly increased phosphorylation of both MEK and ERK in the spinal cord, but not in the DRG. This morphine-induced BRAF kinase overactivity was further supported by direct measurements of BRAF-mediated MEK1 phosphorylation in the spinal cord. Although vemurafenib is widely recognized as a selective inhibitor of BRAF^V600E^, it also potently inhibits WT BRAF (40, 56, 57). We found that vemurafenib did not affect basal BRAF activity in the spinal cord of vehicle-treated rats but effectively reversed morphine-induced BRAF activation, confirming its efficacy in suppressing overactive WT BRAF in neural tissues. Furthermore, morphine-induced MEK phosphorylation was abolished in mice with conditional *Braf* knockout in DRG neurons, providing strong evidence that opioid treatment enhances BRAF activity at primary afferent terminals in the spinal cord, thereby driving downstream MEK/ERK signaling.

A striking finding of our study is that opioid treatment promotes BRAF translocation from DRG neurons to the spinal cord without altering *Braf* mRNA levels in either location. Morphine treatment reduced BRAF protein levels in the DRG while increasing its abundance in spinal cord synaptosomes. In contrast, morphine treatment did not alter total MEK or ERK levels in either the DRG or spinal cord. Conditional *Braf* knockout in DRG neurons not only eliminated BRAF expression in the DRG but also diminished BRAF levels in spinal cord synaptosomes. These findings suggest that a substantial fraction of spinal BRAF originates from DRG neuronal somata and is recruited to their central terminals upon opioid exposure. In support of this interpretation, morphine-induced BRAF recruitment to spinal synaptosomes was abolished by *Braf* ablation in DRG neurons. Such trafficking may be essential for precise spatiotemporal regulation of downstream signaling, particularly MEK/ERK phosphorylation at nociceptor terminals in the spinal dorsal horn. Morphine treatment selectively promotes the translocation of BRAF from DRG neuronal somata to their central terminals, explaining why increased BRAF protein was readily detected in spinal synaptosomes — enriched in primary afferent terminals — but not in whole dorsal spinal tissues. Notably, morphine or fentanyl treatment similarly enhances the anterograde trafficking of Gαq-coupled mGluR5 and α2δ-1 from DRG neurons to their central terminals (16, 19). Together, these findings suggest that the mGluR5-Gαq-PKC and BRAF-MEK-ERK signaling pathways may operate collaboratively within a large protein complex in primary sensory neurons through which opioids modulate spinal synaptic plasticity.

The mechanisms by which opioid treatment promotes BRAF trafficking from the DRG to the spinal cord remain unclear. We found that blocking MORs with FNA eliminated morphine-induced BRAF trafficking to the spinal cord, indicating a critical role for MOR activation. However, we did not detect a direct interaction between MOR and BRAF in either the DRG or spinal cord. Opioid treatment increases PLC and PKC activity in DRG neurons (58), potentially leading to BRAF phosphorylation and subsequent trafficking from DRG neurons to spinal synapses. Motor proteins, such as dyneins and kinesins (59), may also contribute to opioid-induced BRAF transport from the DRG to the spinal cord. While RAF proteins typically activate ERK signaling as homo- and heterodimers in the presence of active RAS (60, 61), activated BRAF appears as monomers in SDS-PAGE gels (62). We detected only BRAF monomers (approximately 85 kDa) in the DRG and spinal cord from both control and morphine-treated rats. Because BRAF dimers are stabilized by salt bridges and hydrogen bonds (63), they may dissociate under denaturing SDS-PAGE conditions.

Our findings reveal that BRAF derived from primary sensory neurons is essential for opioid-induced NMDAR phosphorylation in the spinal cord. Both pharmacological BRAF inhibition and *Braf* knockout in DRG neurons abolished morphine-induced NMDAR phosphorylation in spinal cord synaptosomes, indicating that BRAF activity at primary afferent central terminals is required for this process. Furthermore, BRAF directly interacted with GluN1 in the spinal cord, and this interaction was augmented by morphine treatment. In contrast, BRAF did not associate with GluN1 or MORs in the DRG. Because morphine also promotes ERK association with NMDARs in the spinal cord (18), these findings suggest a direct role of BRAF-ERK signaling in opioid-induced NMDAR phosphorylation. We demonstrated that GluN1 formed protein complexes with BRAF and ERK in human spinal cord tissues, indicating that coupling of NMDARs to the BRAF-ERK pathway is conserved across species. Notably, the GluN1-BRAF complex appeared more abundant in tissue from an opioid overdose donor than from a donor without documented opioid use. Although the central terminals of DRG neurons reside in the spinal dorsal horn, they remain integral components of DRG neurons. Our findings collectively suggest that BRAF physically associates with NMDARs and promotes NMDAR phosphorylation through activated downstream signaling at primary afferent central terminals, underscoring the spatial specificity of BRAF signaling in opioid-induced NMDAR phosphorylation.

Although BRAF was required for morphine-induced GluN1 phosphorylation and physically interacted with GluN1, it remains unresolved whether BRAF directly phosphorylates NMDARs. ERK preferentially phosphorylates serine or threonine residues within serine/threonine-proline (S/TP) motifs (64). While the GluN1 C-terminal tail lacks canonical S/TP motifs, an SP motif (S584-P585) is present within its first cytoplasmic domain, suggesting the possibility of direct ERK-mediated phosphorylation. Opioids also increase PKC activity in the spinal cord (1, 3, 12, 65), and PKC-dependent NMDAR phosphorylation contributes to opioid-induced synaptic trafficking and receptor hyperactivity (3, 12). PKC further links Gαq-coupled mGluR5 signaling to opioid-induced NMDAR hyperactivity in the spinal dorsal horn (20). Although PKC is required for opioid-induced ERK activation in cell lines (23, 66), the precise convergence of the Gαq–PKC and BRAF-MEK-ERK pathways in regulating NMDAR phosphorylation remains to be determined. Because Gαi and Gαq proteins can cooperatively activate ERK (33), and mGluR5, BRAF, and ERK each associate directly with NMDARs (18, 19, 67), it is plausible that opioid-induced BRAF/MEK/ERK signaling phosphorylates NMDARs either directly or indirectly through the Gαq-PKC pathway.

Another notable finding is that BRAF activity is critically involved in opioid-induced synaptic expression of α2δ-1–bound NMDARs in the spinal cord. α2δ-1, originally characterized as a calcium channel subunit, is now recognized as a phospho-binding protein that preferentially interacts with phosphorylated NMDARs (45, 46, 68). Morphine treatment enhances the α2δ-1–NMDAR interaction and synaptic localization of α2δ-1–bound NMDARs at primary afferent central terminals (16). Inhibition of α2δ-1 with gabapentinoids or disruption of the α2δ-1–NMDAR complex suppresses opioid-induced presynaptic NMDAR hyperactivity, hyperalgesia, and tolerance (16). In this study, both pharmacological BRAF inhibition and conditional *Braf* ablation in DRG neurons reversed morphine-induced increases in synaptic expression of α2δ-1–bound NMDARs, supporting BRAF as an upstream regulator of opioid-induced synaptic trafficking of NMDARs in the spinal dorsal horn. Although our study focused on NMDAR-related mechanisms, opioid-induced BRAF activity may also affect other substrates, including MORs and associated G proteins, thereby contributing to reduced opioid analgesic efficacy.

Our study provides compelling evidence that BRAF signaling is a key driver of opioid-induced NMDAR presynaptic hyperactivity in the spinal dorsal horn. NMDAR hyperactivity at primary afferent central terminals mediates opioid-induced hyperalgesia and tolerance (12, 16, 18–20). Increased phosphorylation and synaptic trafficking of α2δ-1–bound NMDARs underlie presynaptic NMDAR hyperactivity in this region (16, 20). We have shown that ERK inhibition blocks opioid-induced presynaptic NMDAR hyperactivity (18). Using a spinal cord slice preparation that preserves native MORs and the synaptic connection between primary afferents and spinal dorsal horn neurons, we found that inhibiting BRAF with vemurafenib or ablating *Braf* in DRG neurons diminished morphine-induced, NMDAR-mediated increases in mEPSC frequency evoked EPSC amplitude, with corresponding changes in EPSC paired-pulse ratio. These results establish BRAF overactivity as a critical upstream driver of opioid-induced presynaptic NMDAR hyperactivity in the spinal cord and underscore the role of BRAF signaling in primary sensory neurons in shaping opioid-induced nociceptive transmission.

Our findings indicate that BRAF in primary sensory neurons is a key mediator of opioid-induced hyperalgesia and tolerance. Opioid-induced presynaptic NMDAR hyperactivity enhances nociceptive input from TRPV1-expressing primary afferents to spinal excitatory neurons, resulting in hyperalgesia (2, 12, 15, 69). Because MOR agonists do not inhibit NMDAR-mediated nociceptive transmission (16, 19, 20), this hyperactivity also contributes to reduced opioid analgesic efficacy and tolerance. We previously showed that spinal MAPK/ERK inhibition attenuates morphine-induced hyperalgesia and tolerance (18). Here, BRAF inhibition with vemurafenib or MEK inhibition with selumetinib produced similar effects, with greater efficacy against hyperalgesia than tolerance. In addition, siRNA-induced *Braf* knockdown or conditional *Braf* ablation in DRG neurons enhanced acute morphine analgesia and attenuated both hyperalgesia and tolerance. Notably, morphine produced greater analgesic efficacy on day 1 in *Braf*-cKO mice and *Braf*-specific siRNA–treated rats than in controls, suggesting that BRAF contributes to the initiation of opioid-induced maladaptive plasticity. These complementary loss-of-function analyses provide strong evidence that BRAF signaling in primary sensory neurons counteracts opioid analgesia and promotes hyperalgesia and tolerance.

In conclusion, our study identifies aberrant BRAF signaling as a key driver of opioid-induced hyperalgesia and tolerance by promoting NMDAR phosphorylation and hyperactivity at nociceptor central terminals in the spinal cord (Supplemental Figure 5). These findings reinforce the concept that signaling events at primary afferent central terminals are critical determinants of opioid analgesic efficacy and adverse plasticity (11, 12, 15, 18–20, 70). Although NMDAR antagonists can alleviate opioid-induced hyperalgesia and tolerance in both preclinical and clinical settings (1, 3, 5, 7), their clinical utility is limited by severe adverse effects. Our findings raise the possibility that BRAF inhibitors, currently used in cancer therapy, may represent an alternative strategy to reduce opioid-induced hyperalgesia and tolerance. Another implication of our study is the potential for opioids to promote tumor growth. Constitutively active BRAF mutants, such as BRAF^V600E^, an oncogenic gain-of-function mutation, drive cancer progression through growth-promoting signaling (36, 37). Although MORs are normally restricted to neurons, they are also expressed in certain cancers, including neuroblastoma (71, 72), and morphine can stimulate the growth of human glioblastoma cells (73). These observations suggest that opioid-induced BRAF activation may inadvertently promote the growth of MOR-expressing cancer cells. Therefore, concurrent use of BRAF inhibitors may not only enhance opioid analgesic efficacy but also potentially mitigate the risk of opioid-associated tumor progression.

## Methods

### Sex as a biological variable.

Sex was not considered as a biological variable because no differences were observed in opioid-induced hyperalgesia and tolerance or agent treatment effects between male and female animals in this study.

### Animal models.

Detailed procedures for animal models and drug administration are provided in the Supplemental Methods.

### Nociceptive behavioral tests.

The pressure withdrawal threshold was measured using a digital Randall-Selitto paw pressure device (#2500, IITC Life Science). The device applied steadily increasing force to the midplantar glabrous surface of the animal’s hindpaw via a pointed end. The force was immediately stopped upon a withdrawal response, and the threshold was recorded (74, 75). The thermal withdrawal latency was measured using a thermal testing apparatus (#390G, IITC Life Science). Animals were placed on a glass surface maintained at 30°C for acclimation. A mobile radiant heat stimulus was applied to the plantar surface of the hindpaw until the animal lifted or licked the hindpaw (2, 69). The time taken for hindpaw withdrawal was recorded as the thermal withdrawal latency.

### Immunoprecipitation and immunoblotting.

Detailed procedures for synaptosome preparation, immunoprecipitation, and immunoblotting are provided in the Supplemental Methods. The specificity of MOR, GluN1, and α2δ-1 antibodies has been validated using knockout mice (11, 16, 19, 20, 48, 76).

### BRAF kinase activity measurement.

BRAF activity in the spinal cord was assessed by measuring phosphorylation of recombinant MEK1 as previously reported (54). Briefly, spinal cord tissues were lysed in IP lysis buffer (#87788, Thermo Fisher Scientific) and were cleared by centrifugation at 10,000 × *g* for 10 minutes at 4°C. Five μg of BRAF antibody (#77622, Cell Signaling Technology) and 100 μL protein G agarose beads (#16-266, MilliporeSigma) were added to the lysates and incubated overnight at 4°C on a slow rotator. The BRAF immunocomplex was washed three times in ice-cold IP lysis buffer to remove unbound material. The immunocomplex was then suspended in ice-cold reaction buffer containing 20 mM Tris (pH 7.4), 20 mM NaCl, 1 mM DTT, 10 mM MgCl_2_, and 1 mM MnCl_2_. The kinase reaction was initiated in the presence of 20 mM ATP and 500 ng of recombinant polyhistidine-tagged MEK1 substrate (#sc-4025, Santa Cruz Biotechnology) incubating at 30°C for 30 minutes with gentle agitation. The reaction was terminated by adding loading buffer containing 1% SDS and 100 mM DTT, followed by boiling for 5 minutes. The eluted samples were then used for immunoblotting analysis of phospho-MEK.

### PCR and immunofluorescence labeling.

Detailed procedures for quantitative PCR and immunofluorescence labeling are provided in the Supplemental Methods.

### Electrophysiological recordings in spinal cord slices.

Detailed procedures for spinal cord slice preparation and recordings are described in the Supplemental Methods. EPSCs from lamina II neurons were recorded in whole-cell voltage-clamp mode at a holding potential of –60 mV, as we described previously (12, 77). EPSCs were evoked by electrical stimulation (0.6 mA, 0.5 ms, and 0.1 Hz) of the dorsal root to elicit glutamate release from primary afferent fibers. Monosynaptic EPSCs were identified by constant latency and absence of conduction failure during 20 Hz stimulation (15, 47). Paired-pulse ratio (PPR) of evoked EPSCs was determined by delivering a pair of stimuli at 50-ms intervals. PPR was calculated as the ratio of the second EPSC amplitude to the first (15, 78). Only one neuron was recorded per slice, and at least four animals were used for each recording protocol.

### Statistics.

Detailed procedures for data and statistical analyses are described in the Supplemental Methods. Data are expressed as mean ± SEM. Sample sizes were based on previous studies and consistent with those generally used in the field (2, 3, 12, 15, 18, 19, 69). Because morphine-induced hyperalgesia, tolerance, and NMDAR hyperactivity were largely overlapping between male and female animals in this and prior studies (12, 19, 20), we combined the data from both groups. However, our study was not specifically powered to detect subtle sex-specific differences. One-way or 2-way ANOVA followed by Tukey’s or Dunnett’s post hoc tests were used to compare more than 2 groups. *P* < 0.05 was considered statistically significant.

### Study approval.

All experimental procedures were approved (approval #0882-RN03 and #1174-RN03) by the Institutional Animal Care and Use Committee of The University of Texas MD Anderson Cancer Center and conducted in accordance with the Guide for the Care and Use of Laboratory Animals (National Institutes of Health).

### Data availability.

Values for all data points in figures are reported in the Supporting Data Values file. Data generated in this study are available from the corresponding author upon reasonable request.

## Author contributions

DJ, HC, YH, and SRC performed research. DJ, SRC, and HLP analyzed data. DJ drafted the paper. SRC and HLP designed research and finalized the paper.

## Conflict of interest

The authors have declared that no conflict of interest exists.

## Funding support

This work is the result of NIH funding, in whole or in part, and is subject to the NIH Public Access Policy. Through acceptance of this federal funding, the NIH has been given a right to make the work publicly available in PubMed Central.

- NIH grants DA041711 and NS101880 to HLP and SRC.
- Pamela and Wayne Garrison Distinguished Chair Endowment to HLP.

## Supplementary Material

Supplemental data

Unedited blot and gel images

Supporting data values

## Version 1 07/14/2026

In-Press Preview

## Version 2 09/01/2026

Electronic publication
