# Supplementary material for: Sensory neuron BRAF mediates opioid-induced hyperalgesia and tolerance via presynaptic NMDA receptor hyperactivity
Source: J Clin Invest. 2026 Jul 14;136(17):e201808. doi: 10.1172/JCI201808 (PMC13528922; doi:10.1172/JCI201808)
Supplement: Supplementary file 1 [file jci-136-201808-s068.pdf]

Supplemental Methods

Animal models

Adult male and female Sprague-Dawley rats (180–250 g) were purchased from Envigo and housed with no more than three rats per cage. *Braf*$^{Flox+/+}$ (Stock#: 006373) (52) and Advillin-Cre (*Avil*$^{iCre/ERT2+}$) (Stock#: 032027) (53) mice were obtained from The Jackson Laboratory. To generate conditional *Braf* knockout in primary sensory neurons, *Braf*$^{Flox+/+}$ mice were crossed with *Avil*$^{iCre/ERT2+}$ mice to produce *Braf*$^{Flox+/-}$::*Avil*$^{iCre/ERT2+}$ offspring, which were then bred with *Braf*$^{Flox+/+}$ mice to obtain *Braf*$^{Flox+/+}$::*Avil*$^{iCre/ERT2+}$ mice. Cre-negative littermates (*Braf*$^{Flox+/+}$::*Avil*$^{iCre/ERT2-}$ or *Braf*$^{Flox+/-}$::*Avil*$^{iCre/ERT2-}$) served as wild-type (WT) control. Genotypes were confirmed using ear biopsies. Conditional *Braf* knockout (*Braf*-cKO) was induced in adult *Braf*$^{Flox+/+}$::*Avil*$^{iCre/ERT2+}$ mice through intraperitoneal (*i.p.*) injection of tamoxifen (75 mg/kg, once daily for 5 days). All mice had a C57BL/6 genetic background and were housed with no more than five mice per cage. Adult male and female mice (8–12 weeks old) were used for final experiments.

Drug and siRNA treatment

Opioid-induced hyperalgesia and tolerance were induced by *i.p.* morphine (#0641-6127-25, West Ward Pharmaceuticals) injection at 10 mg/kg (mice) or 5 mg/kg (rats) twice daily for 7 consecutive days. Vemurafenib (#10618, Cayman Chemical) and selumetinib (#11599, Cayman Chemical) were injected *i.p.* to inhibit BRAF and MEK activity, respectively. β-Funaltrexamine (#72786-10-8, Santa Cruz Biotechnology) was injected intrathecally via lumbar puncture to block MORs in the DRG and spinal cord. For BRAF knockdown in rats, 3 μg *Braf*-specific siRNA (AACCUAUCGUUCGUGUCUUCC) or 3 μg universal negative control siRNA (#SIC001, Sigma-Aldrich) was intrathecally injected daily for 7 or 8 days through an implanted intrathecal catheter (2, 74).

Tissue collection and spinal synaptosome preparation

Frozen lumbar spinal cord tissues from two female donors (aged 44 and 19 years, who died of generalized dystonia and methadone intoxication, respectively) were obtained from the University of Maryland Brain and Tissue Bank. Animals were euthanized by decapitation after deep anesthesia with 5% isoflurane. The DRGs and the dorsal spinal

cords at the L3–L5 level were collected for protein and PCR analyses. Total proteins from the DRG and dorsal spinal cord tissues were extracted using RIPA lysis buffer (MilliporeSigma) containing a protease inhibitor cocktail. To isolate synaptosomal proteins, spinal cord tissues were homogenized in ice-cold homogenization buffer containing 0.32 M sucrose, 10 mM HEPES, 2 mM EDTA (pH 7.4), and a protease inhibitor cocktail. The homogenate was centrifuged at 800 × *g* for 10 min at 4°C to remove insoluble cell debris. The resulting supernatant was further centrifuged at 13,000 × *g* for 20 min to obtain synaptosomes. Synaptosome pellets for immunoblotting were solubilized in RIPA lysis buffer with a protease inhibitor cocktail for 1 hour on ice, followed by centrifugation at 10,000 × *g* for 15 min at 4°C.

Immunoprecipitation and immunoblotting

For immunoprecipitation, proteins were solubilized in immunoprecipitation lysis buffer containing a protease inhibitor cocktail. Samples were incubated at 4°C overnight with protein G beads (MilliporeSigma) and either rabbit anti-pSer antibody (1:100, #AB1603, MilliporeSigma), rabbit anti-GluN1 antibody (1:500, #G8913, MilliporeSigma), mouse anti-BRAF (1:100, #77622, Cell Signaling), normal mouse IgG (#12-371, MilliporeSigma), or normal rabbit IgG (#12-371, MilliporeSigma). After incubation overnight, samples were washed three times with immunoprecipitation lysis buffer. Subsequently, beads were incubated with 1 × NuPage loading buffer supplemented with 100 mM dithiothreitol and 1% sodium dodecyl-sulfate (SDS) for 10 min and boiled for 5 min. Eluted proteins were used for immunoblotting analysis.

Immunoblotting was performed as described previously (18-20). In brief, proteins were separated on 4%–12% SDS NuPage Bis-Tris gels (Thermo Fisher Scientific) and subsequently transferred to polyvinylidene fluoride membranes (MilliporeSigma). Membranes were incubated with primary antibodies overnight at 4°C, followed by incubation with horseradish peroxidase (HRP)-conjugated secondary antibodies (1:5000, rabbit IgG, #7074; 1:5000, mouse IgG, #7076; Cell Signaling) for 1 h at 22°C. For immunoprecipitation samples, HRP-conjugated TrueBlot secondary antibodies were used (1:5,000, rabbit IgG, #18-8816-31; 1:5,000, mouse IgG, #18-8817-31; Rockland Immunochemicals). Membranes were thoroughly washed after each antibody incubation. Immunoblots were developed using a chemiluminescence kit or enhanced chemiluminescence kit (Thermo Fisher Scientific), visualized with an Odyssey Fc Imager (LI-COR Biosciences) and quantified using ImageJ software.

Primary antibodies included rabbit anti-GluN1 (#G8913, MilliporeSigma), rabbit anti-MOR (#AB5511, MilliporeSigma), rabbit anti-BRAF (#14814, Cell Signaling Technology), rabbit anti-phospho-MEK1/2 at Ser217/Ser221 (#9154, Cell Signaling Technology), rabbit anti-phospho-ERK1/2 (#4370, Cell Signaling Technology), rabbit anti-ERK1/2 (#4695, Cell Signaling Technology), rabbit anti-phospho-Serine (pSer, #AB1603, MilliporeSigma), rabbit anti-GAPDH (#SAB1412855, Cell Signaling Technology), mouse anti-MEK1/2 (#SC81504, Santa Cruz Biotechnology), mouse anti-β-actin (catalog #3370, Cell Signaling Technology), mouse anti-PSD95 (#MABN1190, MilliporeSigma), mouse anti-α2δ-1 (#SC-271697, Santa Cruz Biotechnology). The specificity of MOR, GluN1, and α2δ-1 antibodies has been validated using knockout mice (11, 16, 19, 20, 48, 76). Protein band intensity was normalized to PSD95 or β-actin on the same membranes.

Quantitative PCR

Total RNA was extracted from the DRG and dorsal spinal cord tissues with a Trizol reagent (#15596026, Thermo Fisher Scientific) according to the manufacturer's instruction. First-strand cDNA was synthesized using the RevertAid RT Reverse Transcription Kit (#K1691, Thermo Fisher Scientific). Quantitative real-time PCR was performed with the SYBR GreenER qPCR SuperMix Universal kit (#01143681, Thermo Fisher Scientific) on a Quant Studio 7 Flex Real-Time PCR System (Applied Biosystems). Thermal cycling conditions were initial denaturation at 95°C for 5 min, followed by 40 cycles at 95°C for 15 sec, 60°C for 15 s, and 72°C for 40 s. Primers specific for rat *Braf* (sequence ID: NM_001427466.2) were CAAACATCAACAACAGGGATCAG (forward) and GGAGAAGCACAAGCATACAG (reverse). Primers for rat β-actin (sequence ID: NM_031144.3) were TACGTAGCCATCCAGGCTGTG (forward) and CAGCTCATAGCTCTTCTCCAG (reverse). Results are expressed as relative expression compared to control groups after normalizing to β-actin using the comparative $^{\Delta\Delta}$CT method.

Immunofluorescence labeling

Adult rats were deeply anesthetized via intraperitoneal injection of Beuthanasia-D (0.1 ml/kg) and transcardially perfused with sterile normal saline followed by 4% paraformaldehyde. Lumbar DRGs and spinal cord were dissected, post-fixed in the same fixative for 2 h, and cryoprotected overnight in 30% sucrose in 0.1 M Tris-buffered

saline (TBS). Tissue sections (25 µm thick) were cut using a Leica CM1860 cryostat and collected in TBS. Before antibody incubation, sections were rinsed in TBS, permeabilized with 0.1% Triton X-100 for 15 min, and blocked with TNB blocking buffer (#K1050-100-300, Apexbio) for 1 h at 22°C. For double immunofluorescence labeling of BRAF with NeuN, NF200, or CGRP, sections were incubated overnight at 4°C in TNB buffer with a mixture of rabbit anti-BRAF antibody (1:50) with either mouse anti-NeuN (1:300; #ab104224, Abcam), mouse anti-NF200 (1:150, #N0142, Sigma), or goat anti-CGRP (1:150, #ab36001, Abcam). The sections were then incubated for 2 h at 22°C with appropriate secondary antibodies diluted in TNB buffer: Alexa Fluor 488–conjugated donkey anti-rabbit IgG (1:300; #A-21206, Thermo Fisher Scientific), Alexa Fluor 647–conjugated goat anti-mouse IgG (1:300; #A31571, Thermo Fisher Scientific), or Alexa Fluor 592–conjugated donkey anti-goat IgG (1:300; #708-585-147, Jackson ImmunoResearch).

For double labeling of BRAF and IB4, sections were first incubated with rabbit anti-BRAF antibody followed by Alexa Fluor 488–conjugated donkey anti-rabbit IgG. Sections were then incubated for 1 h at 22°C with Alexa Fluor 594–conjugated IB4 (1:500, #I21413, Thermo Fisher Scientific). After thorough rinsing in TBS, sections were mounted on slides, air-dried, and coverslipped using antifade mounting medium. Images were acquired using a fluorescence microscope and a Zeiss confocal laser-scanning microscope. For quantitative analysis of labeled DRG neurons, 10 randomly selected high-magnification (40×) images were obtained from four mice per group. Quantification was performed using the Cell Counter plugin in NIH ImageJ.

Electrophysiological recordings in spinal cord slices

Animals were anesthetized with 3% isoflurane, and lumbar spinal cords at the L3–L5 levels were quickly removed via laminectomy. Animals were then euthanized by inhalation of 5% isoflurane followed by rapid decapitation. The spinal cords were immediately placed in ice-cold artificial cerebrospinal fluid presaturated with 95% O_2 and 5% CO_2, containing (in mM): 25 glucose, 234 sucrose, 3.6 KCl, 26 $NaHCO_3$, 1.2 NaH_2PO_4, 2.5 $CaCl_2$, and 1.2 $MgCl_2$. The tissue was glued onto the stage of a vibratome and sectioned into 400 µm-thick transverse slices. Slices were incubated in Krebs solution containing (in mM): 11 glucose, 117 NaCl, 3.6 KCl, 25 $NaHCO_3$, 1.2 NaH_2PO_4, 2.5 $CaCl_2$, and 1.2 $MgCl_2$ (gassed with 95% O_2 and 5% CO_2) at 34°C for at least 1 hour before recordings. Slices were

then transferred to a recording chamber with continuous perfusion of oxygenated Krebs solution (3 mL/min) at 34°C.

Neurons in the lamina II outer layer were identified using infrared illumination and differential interference contrast under a microscope. EPSCs from these neurons were recorded in whole-cell voltage-clamp mode at a holding potential of –60 mV, as we described previously (12, 77). Recording electrodes (4–7 MΩ) were filled with internal solution containing (in mM): 135 K-gluconate, 5 KCl, 2 $MgCl_2$, 0.5 $CaCl_2$, 5 EGTA, 5 HEPES, 0.5 Na_2-GTP, 5 Mg-ATP, and 10 lidocaine N-ethyl bromide (QX314; 280–300 mOsm, pH 7.3). QX314 was included in the pipette recording solution to suppress postsynaptic neuronal firing. mEPSCs were recorded in the presence of 0.5 μM tetrodotoxin.

EPSCs were evoked by electrical stimulation (0.6 mA, 0.5 ms, and 0.1 Hz) of the dorsal root to elicit glutamate release from primary afferent fibers. Monosynaptic EPSCs were identified by constant latency and absence of conduction failure during 20 Hz stimulation (15, 47). Paired-pulse ratio (PPR) of evoked EPSCs was determined by delivering a pair of stimuli at 50-ms intervals. PPR was calculated as the ratio of the second EPSC amplitude to the first (15, 78).

Signals were processed using an amplifier (MultiClamp700B; Molecular Devices), filtered at 1–2 kHz, and digitized at 10 kHz. Recordings were discontinued if the input resistance changed by more than 15% during the session. Only one neuron was recorded per slice, and at least four animals were used for each recording protocol. AP5 and tetrodotoxin were purchased from Hello Bio Inc. Drugs were diluted in artificial cerebrospinal fluid immediately before use and delivered at their final concentrations via syringe pumps.

Data and statistical analyses

Data are expressed as means ± SEM. Sample sizes were based on previous studies and consistent with those generally used in the field (2, 3, 12, 15, 18, 19, 69). Animals were randomly assigned (1:1 allocation) to control and treatment groups as they became available. Because morphine-induced hyperalgesia, tolerance, and NMDAR hyperactivity were largely overlapping between male and female animals in this and prior studies (12, 19, 20), we combined the data from both groups. However, our study was not specifically powered to detect subtle sex-specific differences. Investigators performing behavioral (DJ) and electrophysiological (HC, YH, and SRC) experiments

were blinded to genotype and treatment. The amplitude and frequency of mEPSCs were analyzed using a peak detection program (MiniAnalysis, Synaptosoft). Cumulative distributions of the amplitude and interevent interval of mEPSCs were compared using the Komogorov-Smirnov test. Evoked EPSCs and PPR were analyzed using Clampfit 10.0 (Molecular Devices), and the amplitude of EPSCs was quantified by averaging 6 consecutive currents. Two-tailed Student's t tests were used for comparisons between two groups. One-way or two-way analysis of variance (ANOVA) followed by Tukey's or Dunnett's post hoc tests were used to compare more than two groups. Statistical analyses were performed using Prism software (version 10, GraphPad Software Inc). $P < 0.05$ was considered statistically significant.

Supplemental Figures

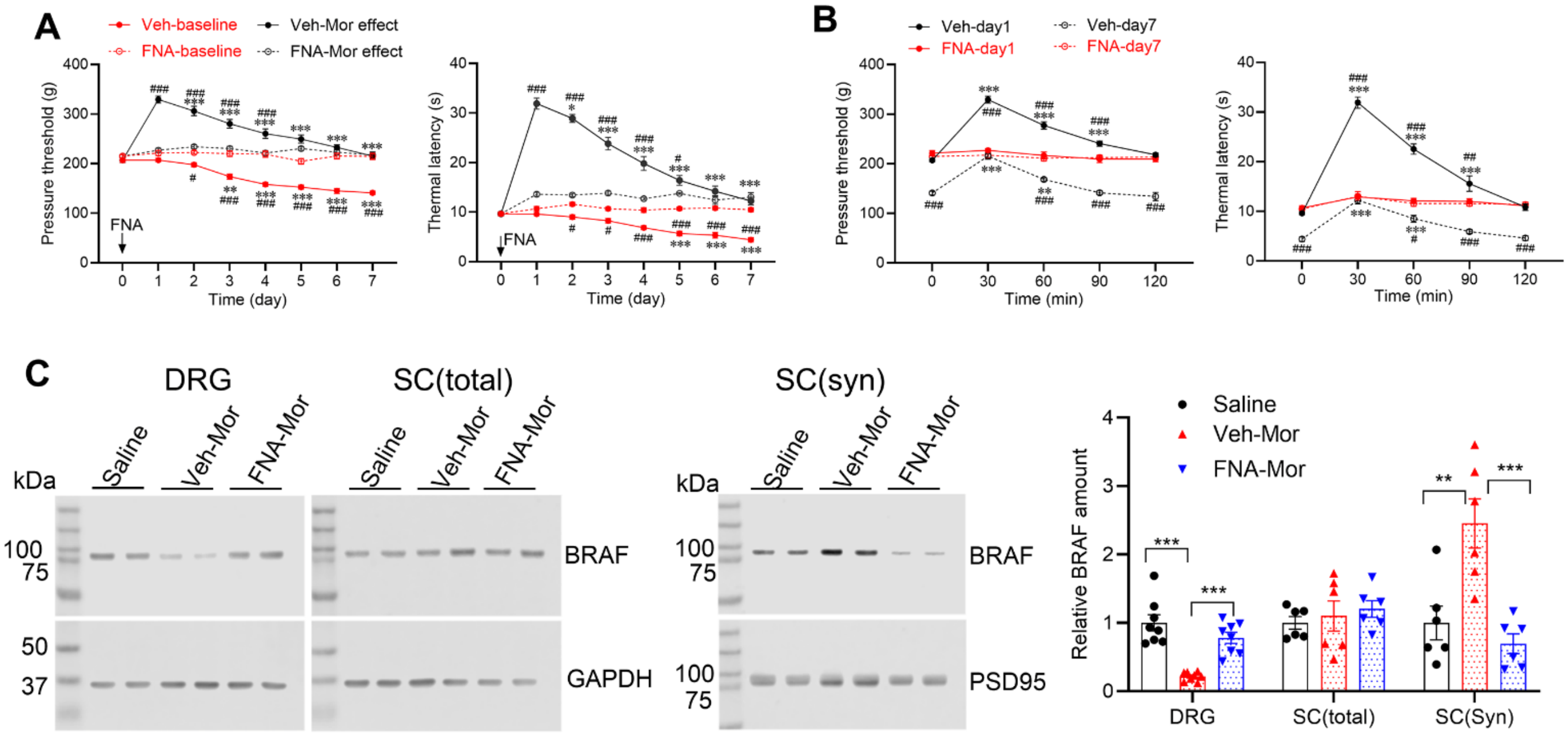

Figure S1. Morphine treatment induces hyperalgesia, tolerance, and BRAF translocation from the DRG to spinal cord via μ-opioid receptors. (**A**) Time course of morphine-induced hyperalgesia and tolerance in rats treated with β-funaltrexamine (FNA) + morphine (Mor) or vehicle (Veh) + Mor. Rats received a single intrathecal injection of 10 μg of FNA or Veh followed by treatment of morphine (5 mg/kg) twice daily for 7 days. Nociceptive thresholds and latency were tested before (baseline) and 30 min after the first morphine injection daily (n = 8 per group). (**B**) Time course of the acute analgesic effect of morphine in rats treated with FNA + Mor or Veh + Mor. Nociceptive thresholds and latency were tested after the first morphine injection on day 1 and day 7 (n = 8 per group). (**C**) Representative immunoblot images and quantification show BRAF protein expression in the DRG (n = 8) and spinal cord (SC, n = 6) from rats treated with saline, FNA + Mor, or Veh + Mor. Total and synaptosomal (Syn) proteins were extracted for immunoblotting analysis after behavioral tests. GAPDH and PSD95 were used as loading controls. Data are expressed as means ± SEM. In Panels **A** and **B**, *P < 0.05, **P < 0.01, ***P < 0.001 vs. respective values at day 1 or time (min) 0. $^{\#}$P < 0.05, $^{\#\#\#}$P < 0.001 vs. respective FNA+Mor groups at the same time point (two-way ANOVA followed by Tukey's post hoc test). In Panel **C**, **p < 0.01, ***p < 0.001 (one-way ANOVA followed by Tukey's post hoc test).

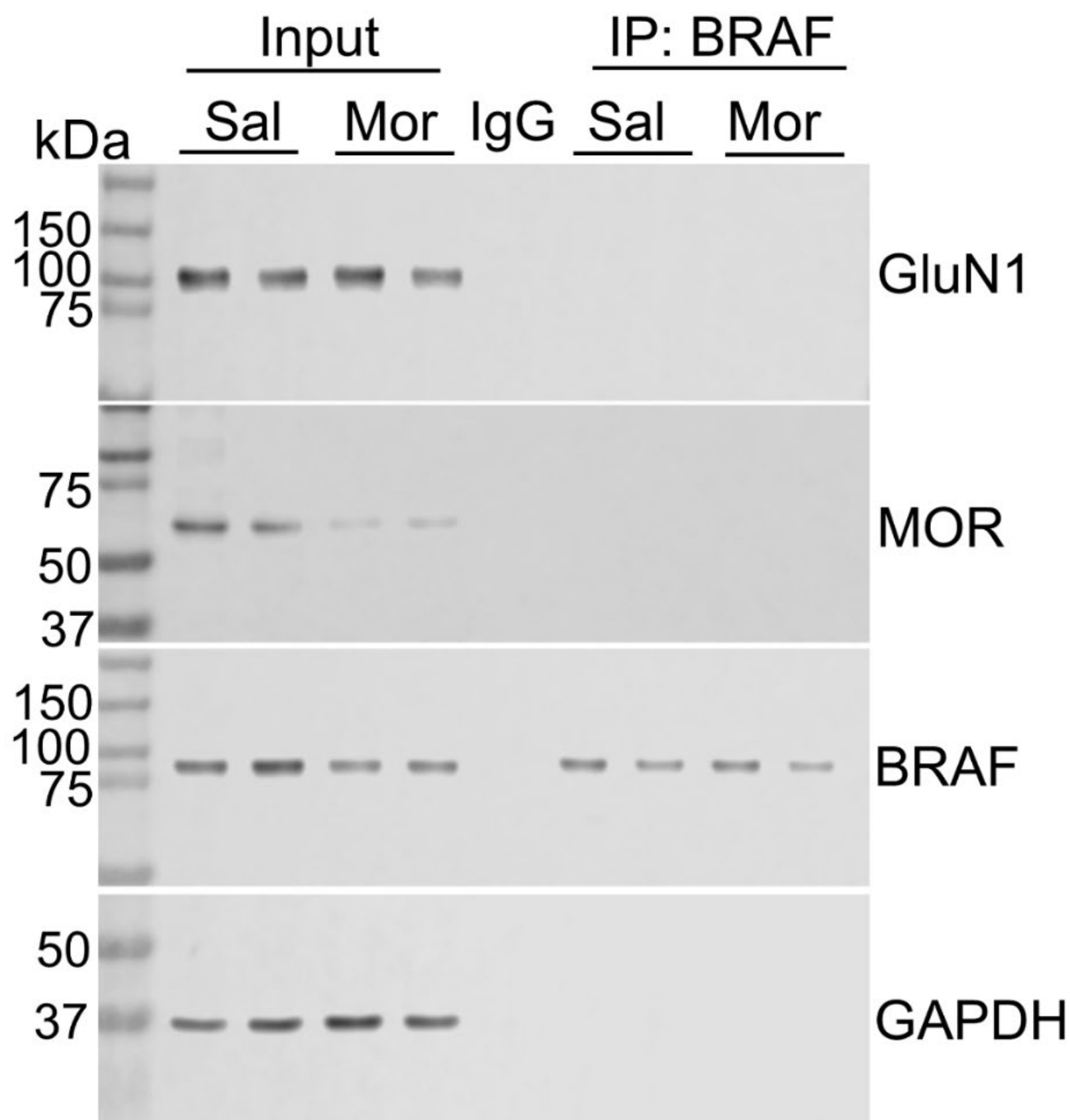

Figure S2. BRAF does not interact with GluN1 or MOR in the rat DRG. Representative immunoblot images show neither GluN1 nor μ-opioid receptor (MOR) was detected in BRAF immunoprecipitates in the protein extracts from the DRG of rats treated with saline (Sal) or morphine (Mor). Proteins were immunoprecipitated with a BRAF antibody or IgG. Immunoblotting was then performed using GluN1, MOR, BRAF, and GAPDH antibodies. IgG and input samples (tissue lysates without immunoprecipitation) served as negative and positive controls, respectively. Comparable immunoblot results were obtained from two independent experiments.

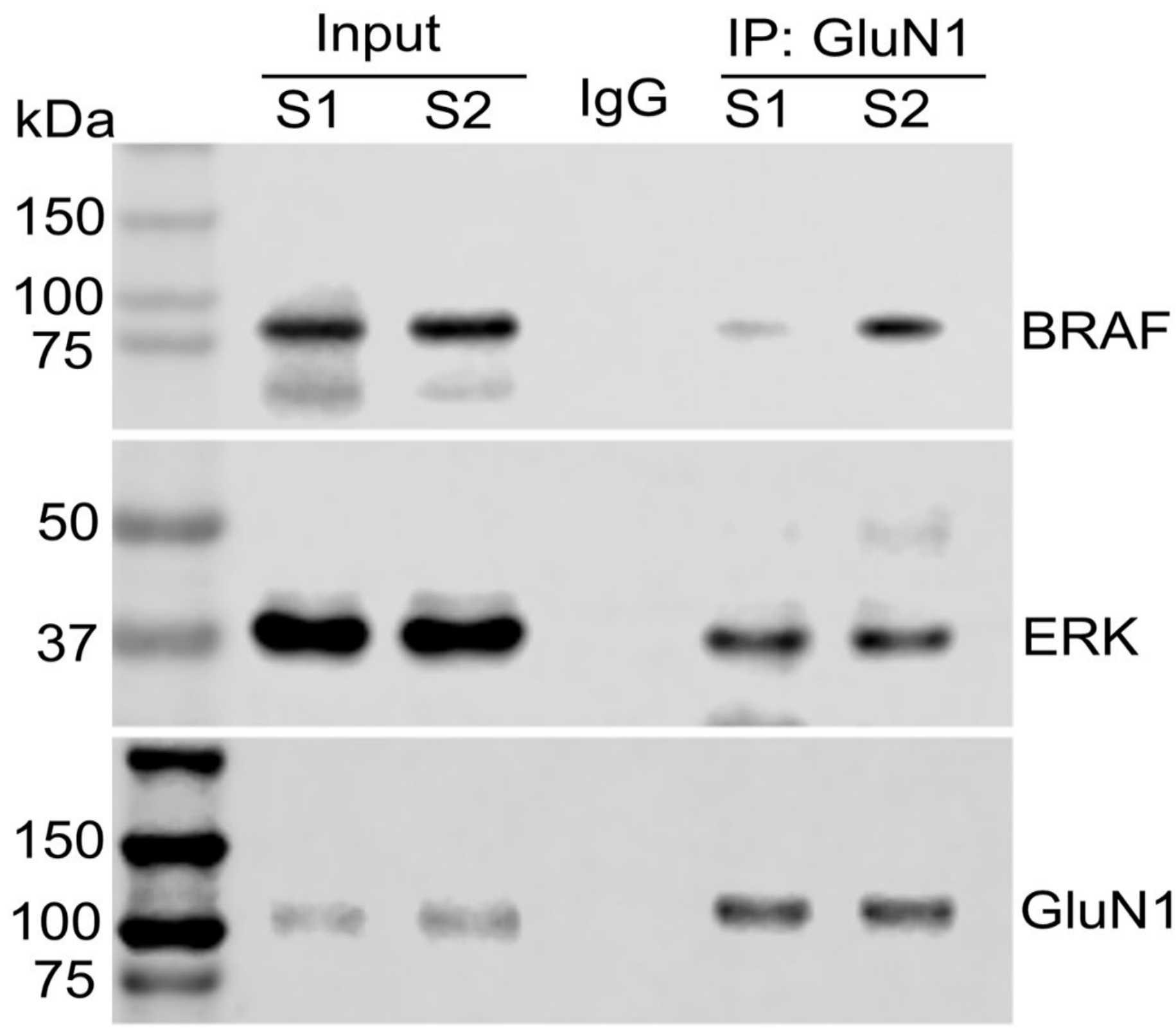

Figure S3. Direct association of NMDARs with BRAF and ERK in the human spinal cord. Representative immunoblot images show GluN1 interacted with BRAF and ERK1/2 in the total protein extracts of human lumbar spinal cord tissues. Proteins were immunoprecipitated with a GluN1 antibody or IgG. Immunoblotting was then performed using GluN1, BRAF, and ERK1/2 antibodies. IgG and input samples (tissue lysates without immunoprecipitation) served as negative and positive controls, respectively. Comparable immunoblot results were obtained from two independent experiments. S1 refers to the donor who died of generalized dystonia, and S2 refers to the donor who died of methadone intoxication.

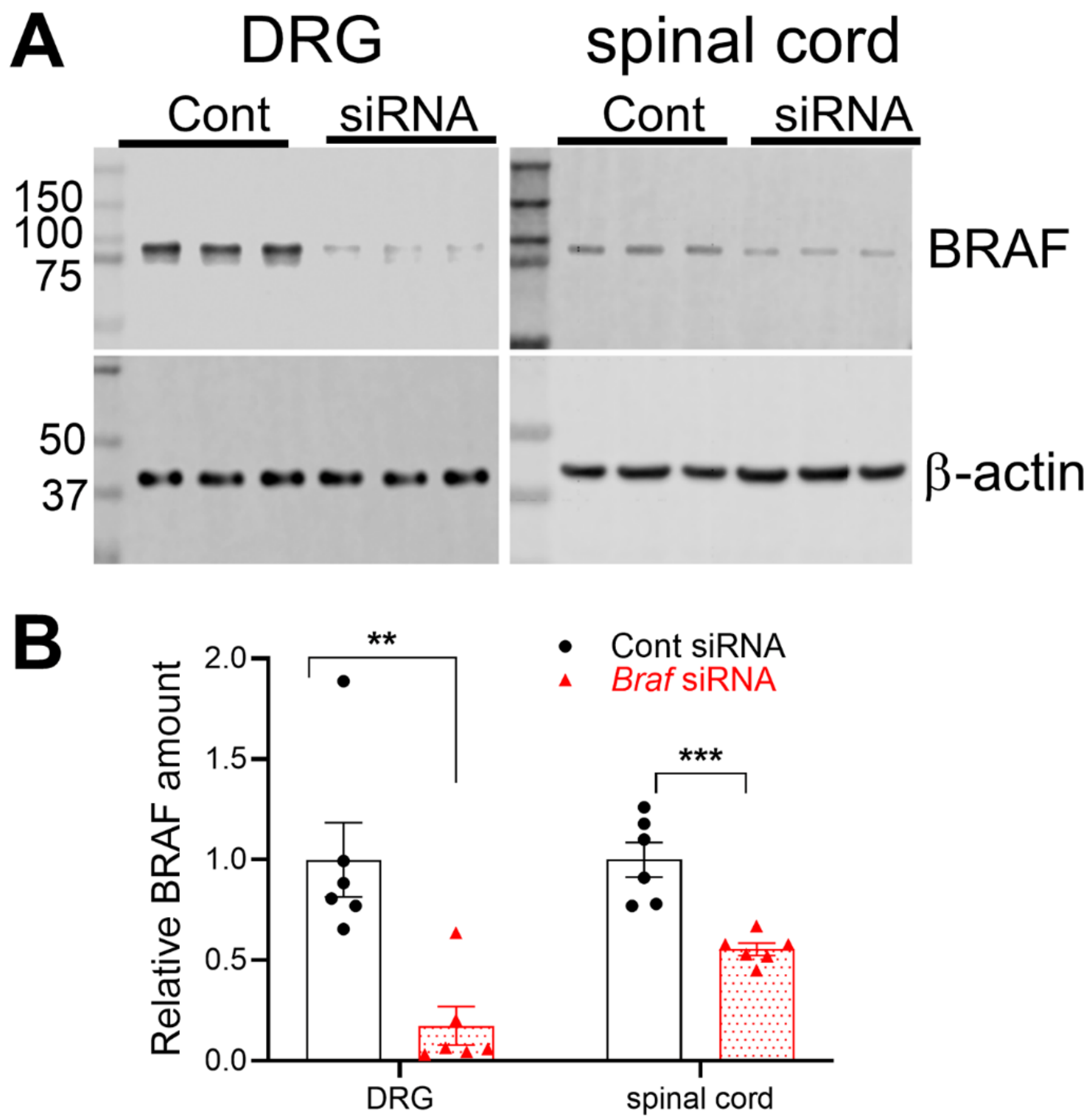

Figure S4. siRNA-mediated BRAF knockdown in the DRG and spinal cord. (A and B) Representative immunoblot images (**A**) and quantification (**B**) show the amount of BRAF protein in the DRG and spinal cord tissues from rats treated with intrathecal injection of 3 µg *Braf*-specific siRNA or control (Cont) siRNA for 7 days (n = 6 rats per group). β-actin was used as a loading control. Data are expressed as means ± SEM. $^{**}P < 0.01$, $^{***}P < 0.001$ (two-tailed Student's t test).

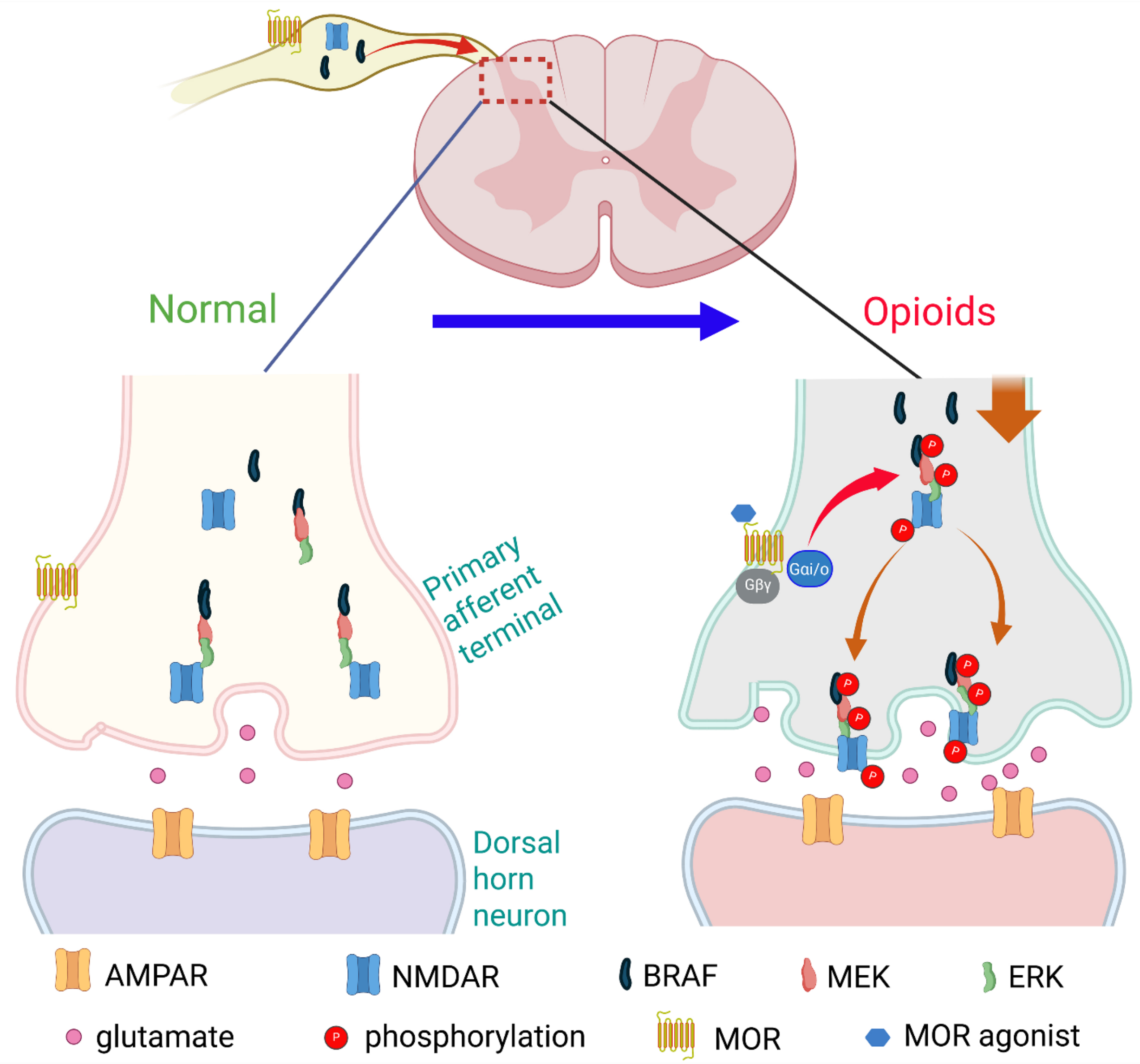

Figure S5. Schematic representation of BRAF recruitment and the signaling cascade underlying opioid-induced potentiation of synaptic NMDAR expression at primary afferent central terminals. Under basal conditions, BRAF, MEK, ERK, and NMDARs exhibit minimal phosphorylation and limited interaction within primary afferent terminals. Opioid treatment promotes BRAF trafficking from DRG neuronal somata to their central terminals in the spinal dorsal horn. At these terminals, the phosphorylated BRAF/MEK/ERK signaling complex interacts with and enhances phosphorylation of NMDARs, thereby increasing their synaptic expression and activity. This opioid-induced enhancement of synaptic glutamate release amplifies nociceptive input to spinal dorsal horn neurons, contributing to hyperalgesia and reduced opioid analgesic efficacy.
