# Supplementary material for: Sensory neuron BRAF mediates opioid-induced hyperalgesia and tolerance via presynaptic NMDA receptor hyperactivity
Source: J Clin Invest. 2026 Jul 14;136(17):e201808. doi: 10.1172/JCI201808 (PMC13528922; doi:10.1172/JCI201808)

Fig. 2A Day 3

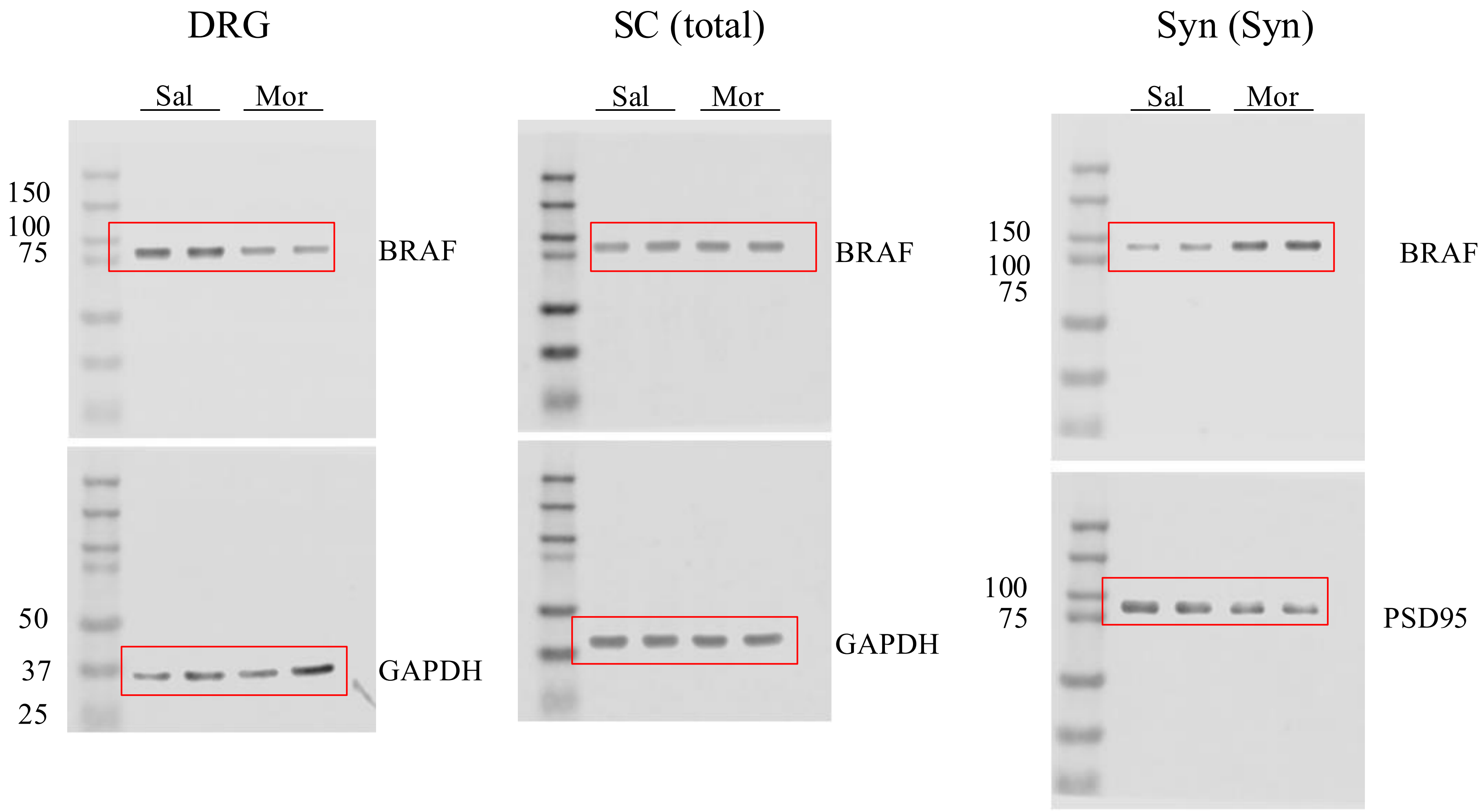
DRG
SC (total)
Syn (Syn)
Sal
Mor
Sal
Mor
Sal
Mor
150
100
75
BRAF
50
37
25
GAPDH
BRAF
GAPDH
150
100
75
BRAF
100
75
PSD95

Fig. 2B Day 7

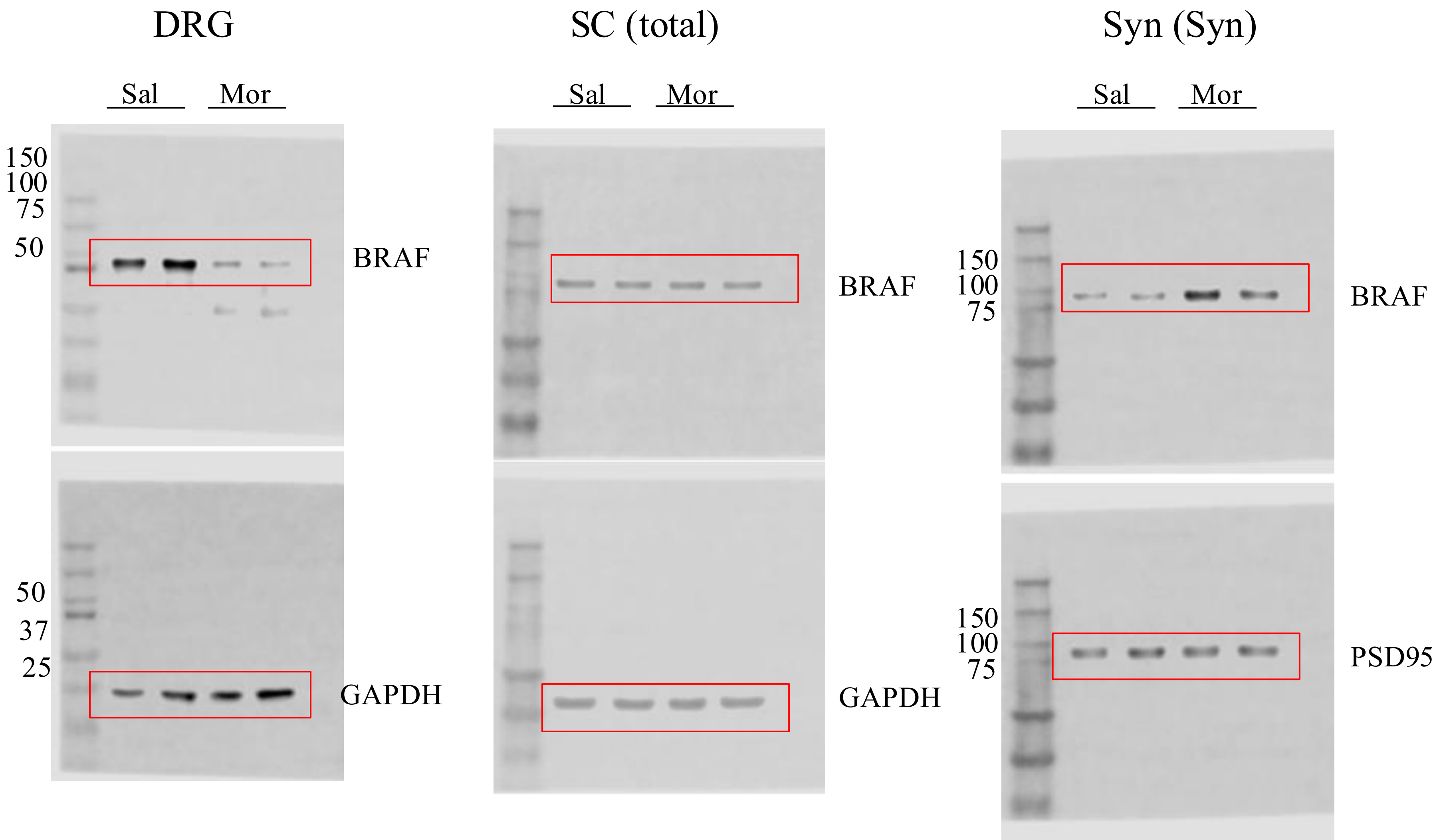
DRG
SC (total)
Syn (Syn)
Sal
Mor
Sal
Mor
Sal
Mor
150
100
75
50
BRAF
BRAF
150
100
75
BRAF
50
37
25
GAPDH
GAPDH
150
100
75
PSD95

Fig. 3A pMEK

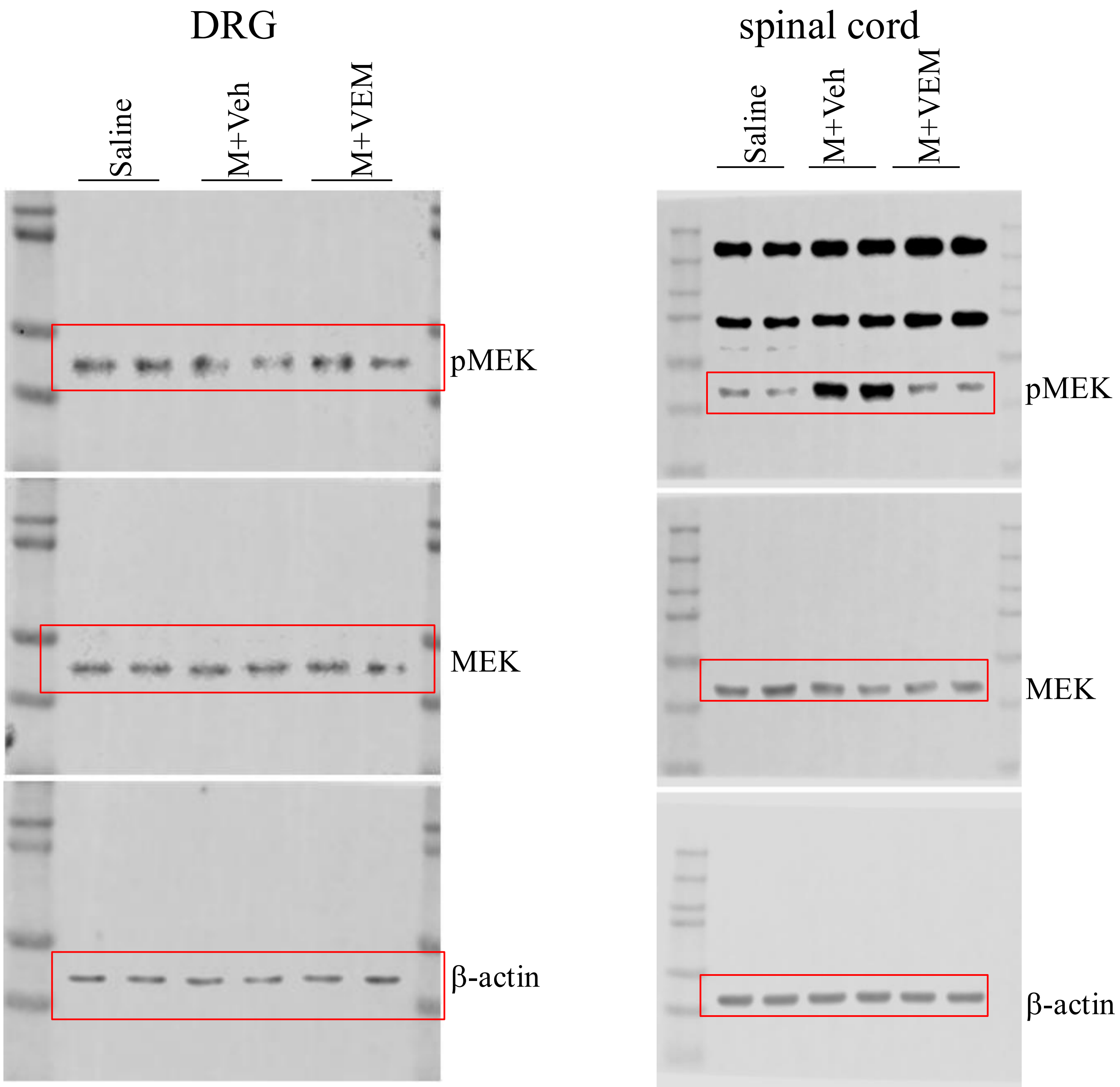

DRG
Saline
M+Veh
M+VEM
pMEK
MEK
β-actin
spinal cord
Saline
M+Veh
M+VEM
pMEK
MEK
β-actin

Fig. 3B pMEK by VEM alone

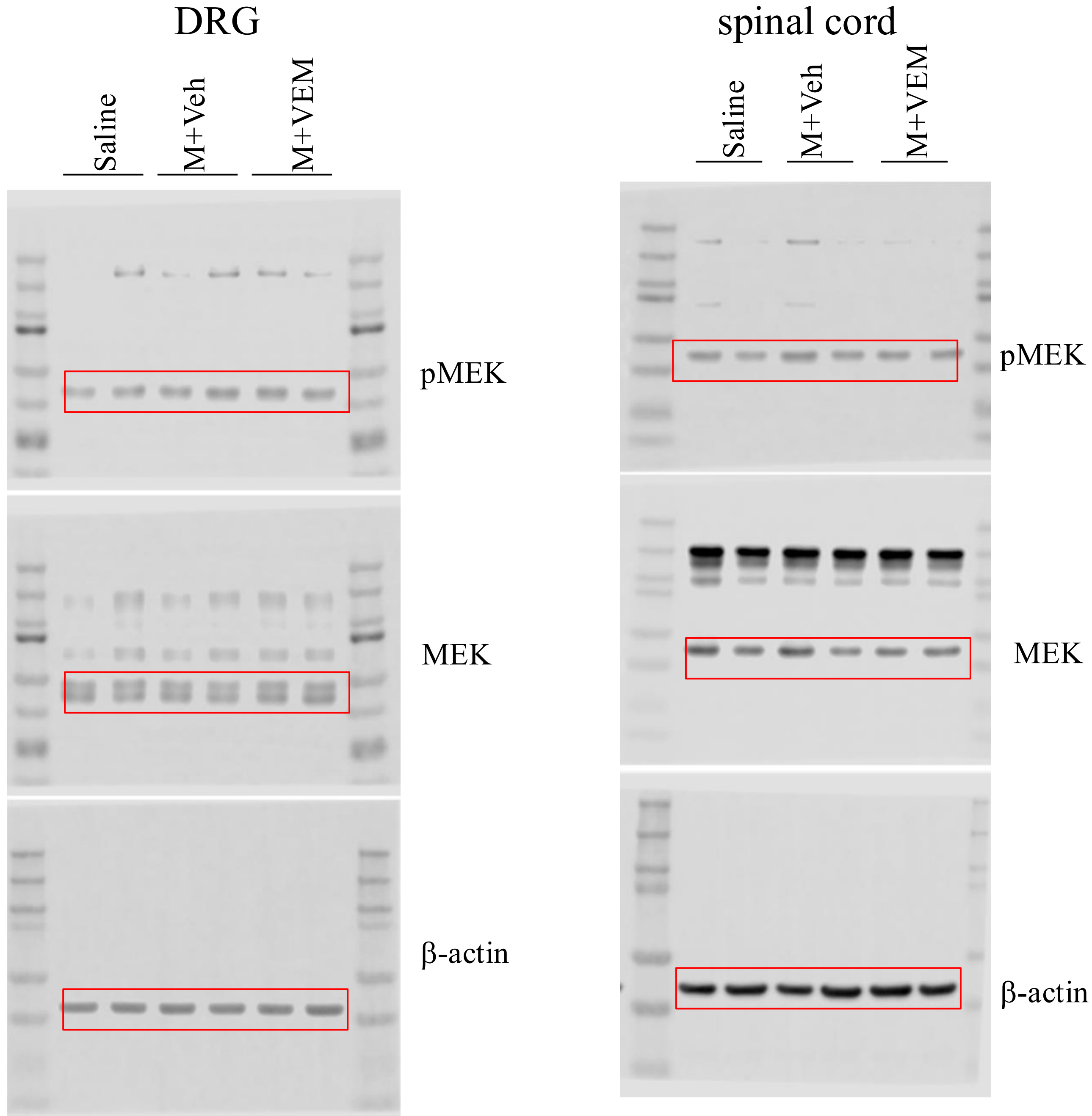

Fig. 3C pERK

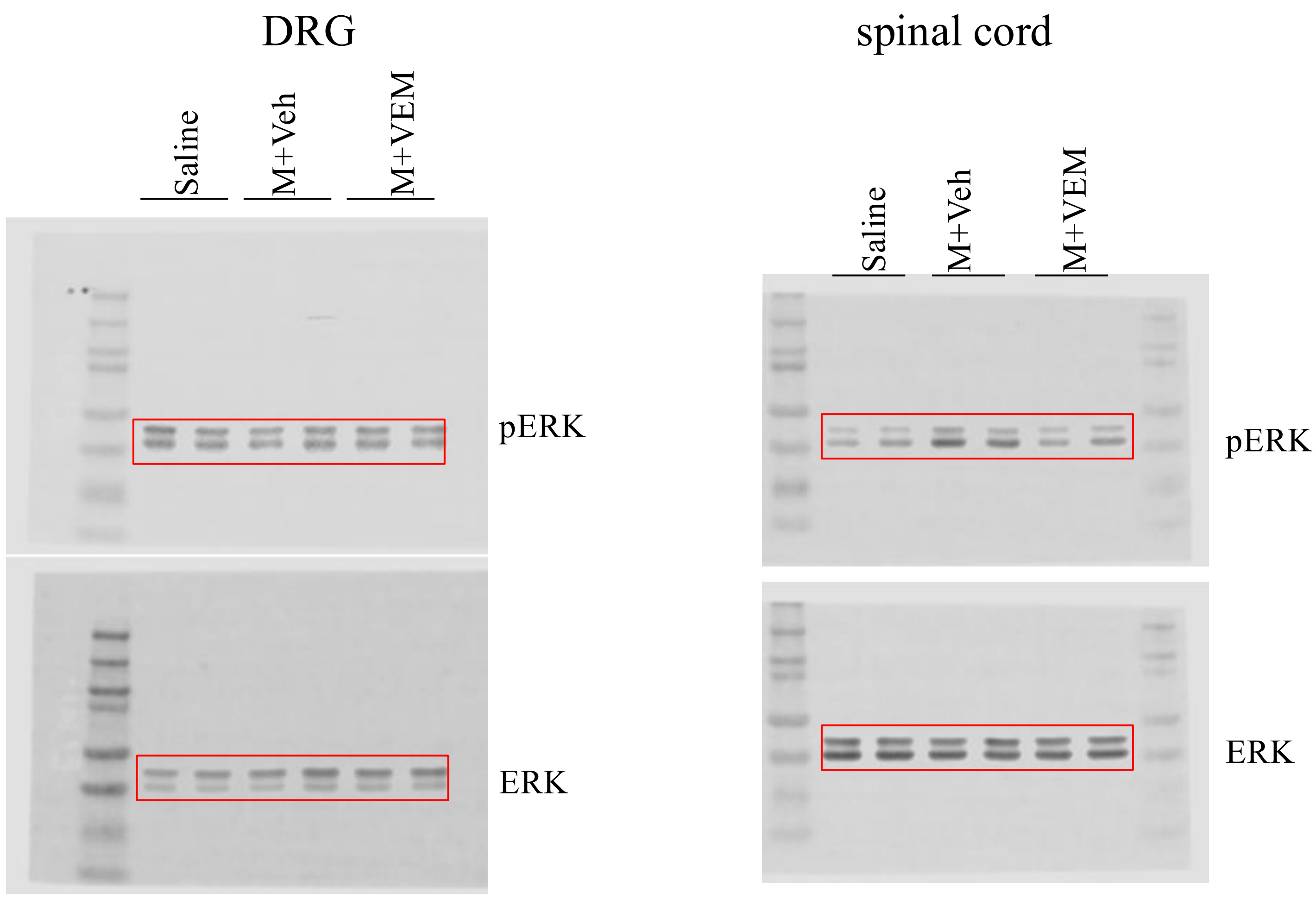

DRG
Saline
M+Veh
M+VEM
pERK
ERK
spinal cord
Saline
M+Veh
M+VEM
pERK
ERK

Fig. 3D pMEK by BRAF-IP

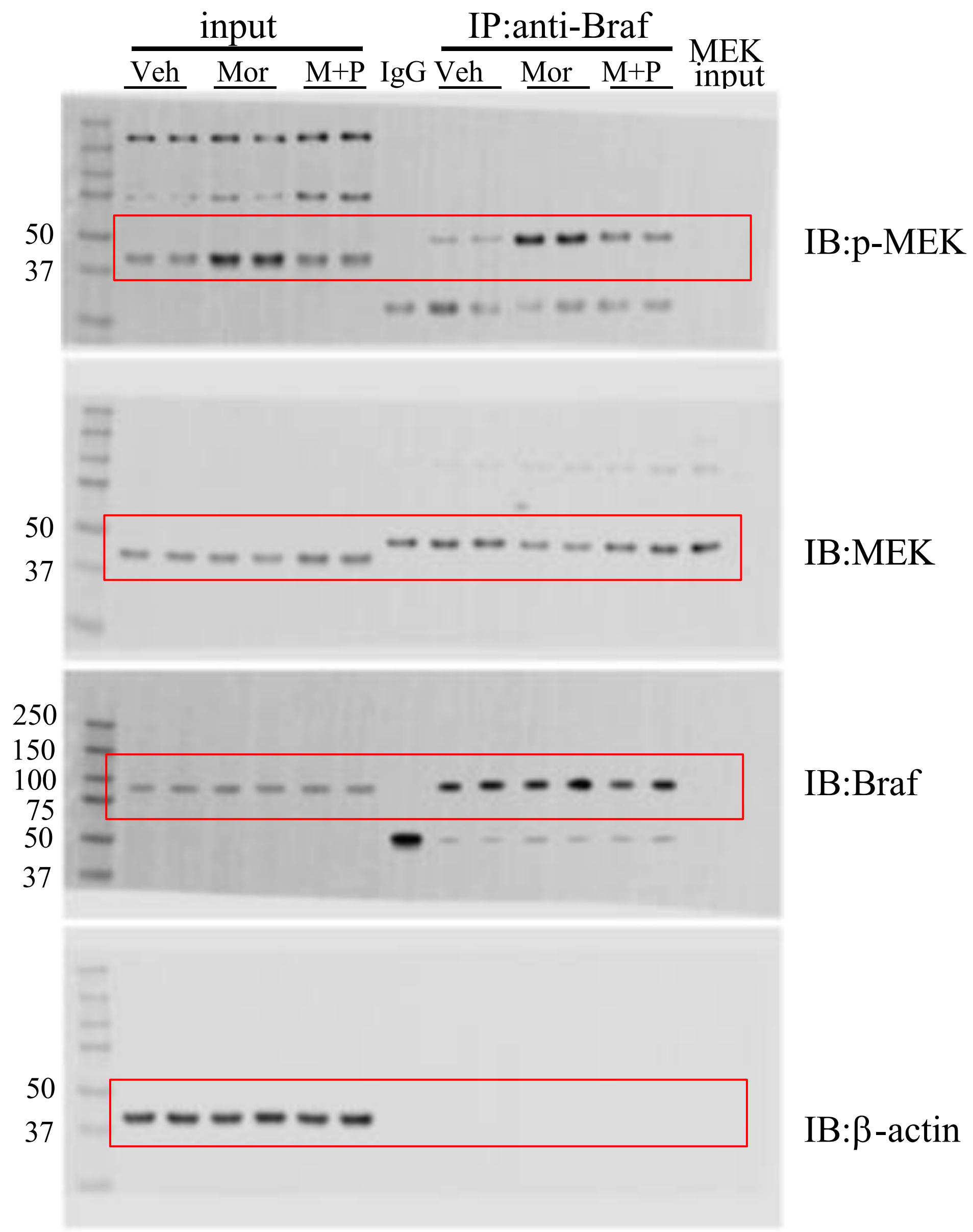

Fig. 4A pSer phosphorylation

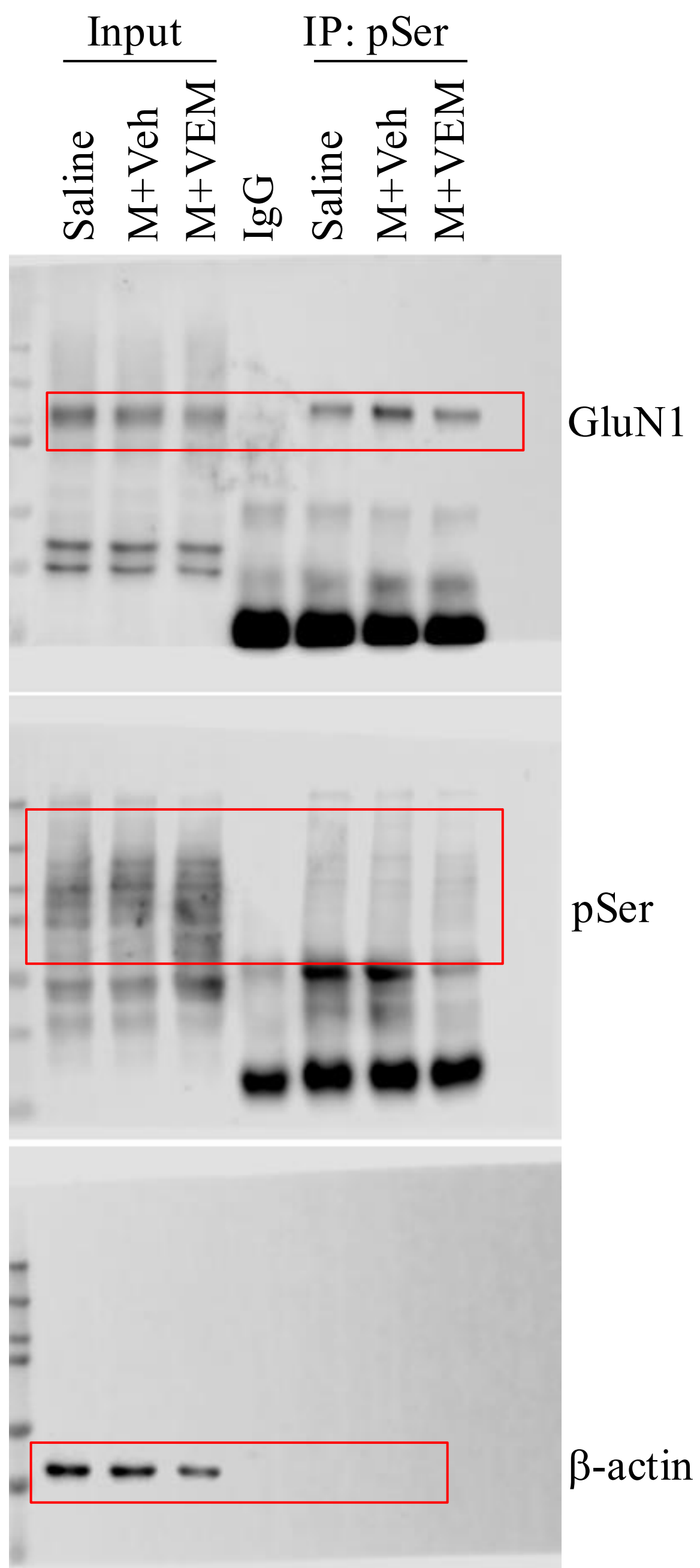

Fig. 4B BRAF-IP

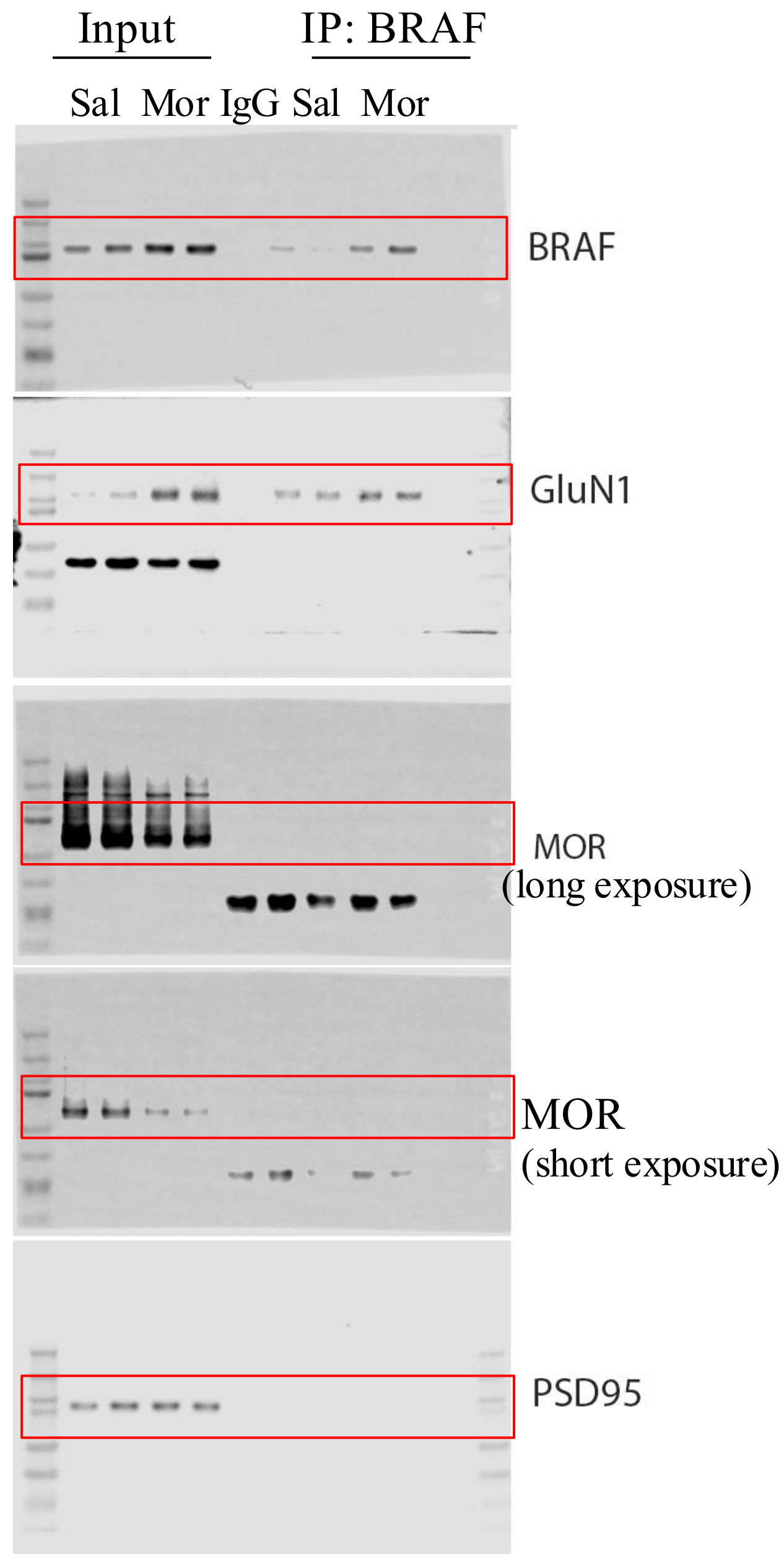

Fig. 4C GluN1-IP

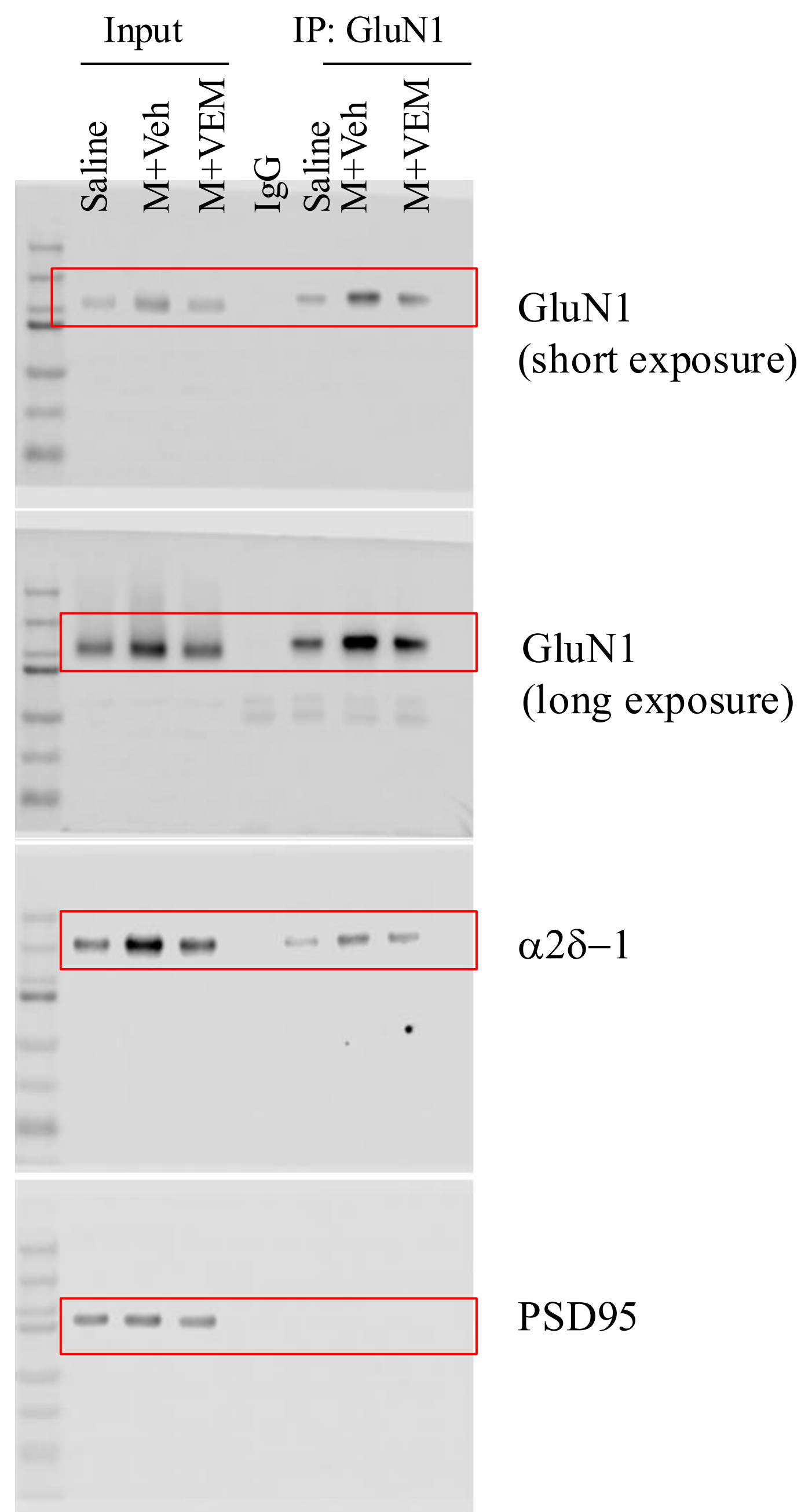

Fig. 8A

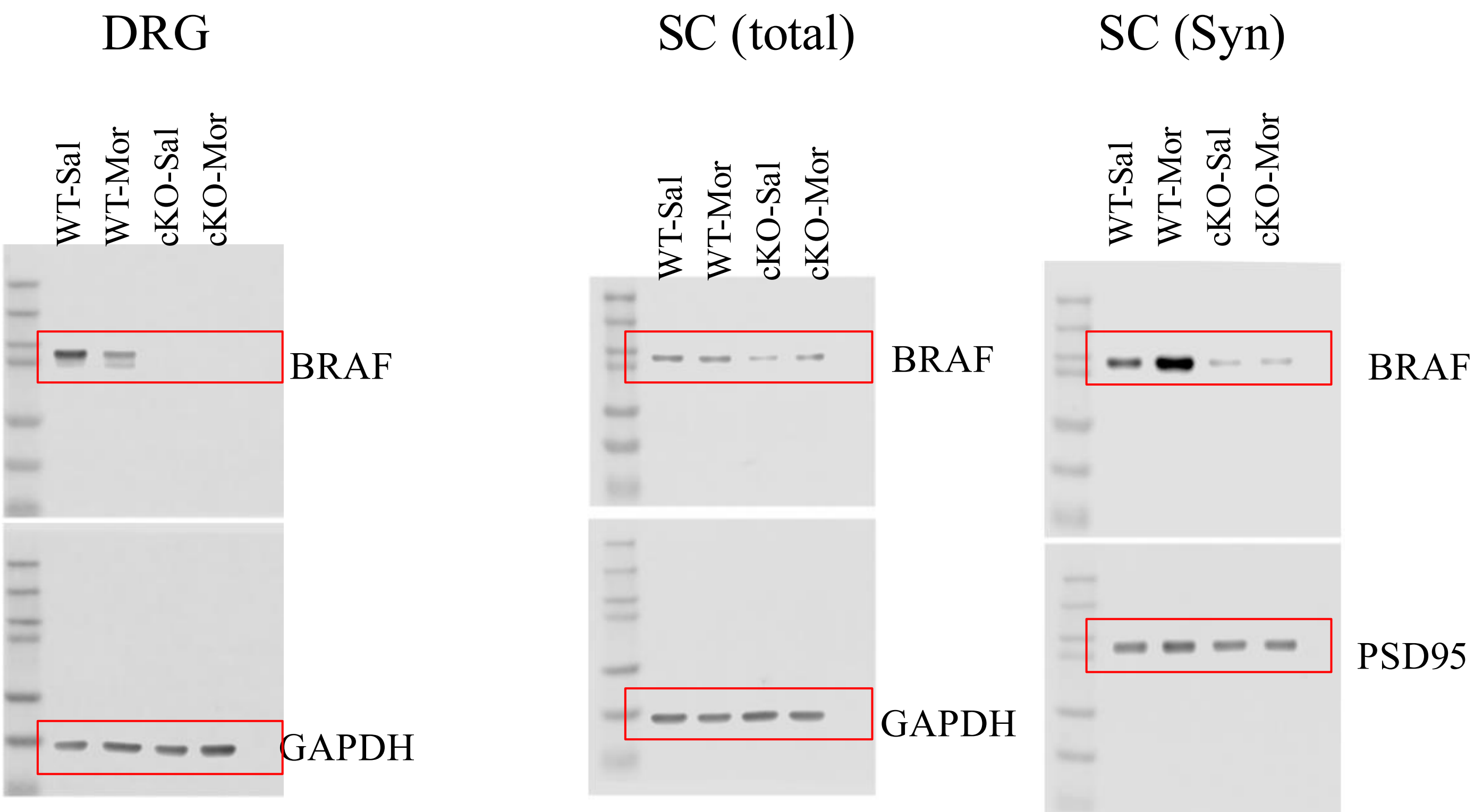
DRG
SC (total)
SC (Syn)
WT-Sal
WT-Mor
cKO-Sal
cKO-Mor
BRAF
GAPDH
BRAF
GAPDH
BRAF
PSD95

Fig. 8B

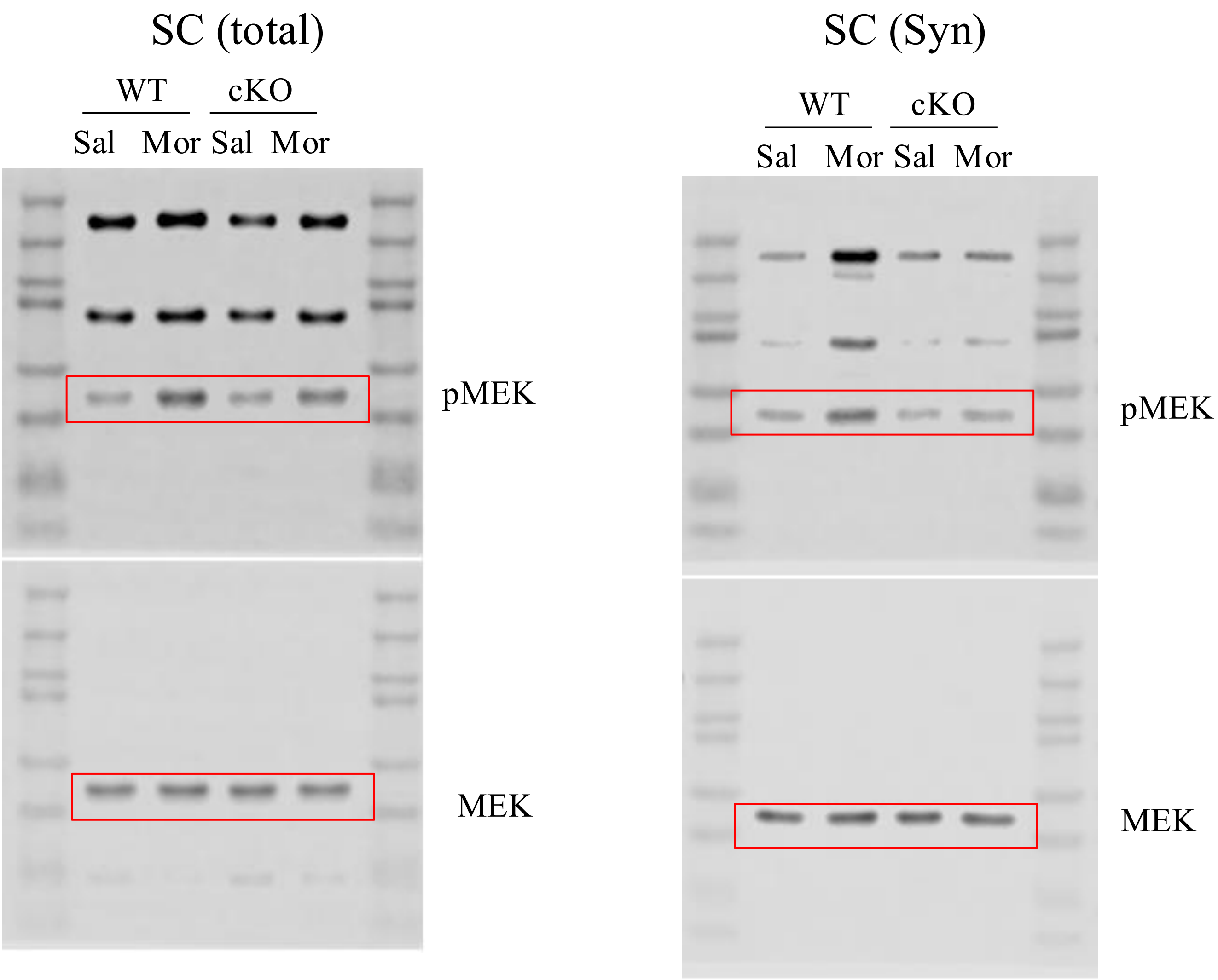
SC (total)
WT
cKO
Sal
Mor
Sal
Mor
pMEK
MEK
SC (Syn)
WT
cKO
Sal
Mor
Sal
Mor
pMEK
MEK

Fig. 9A

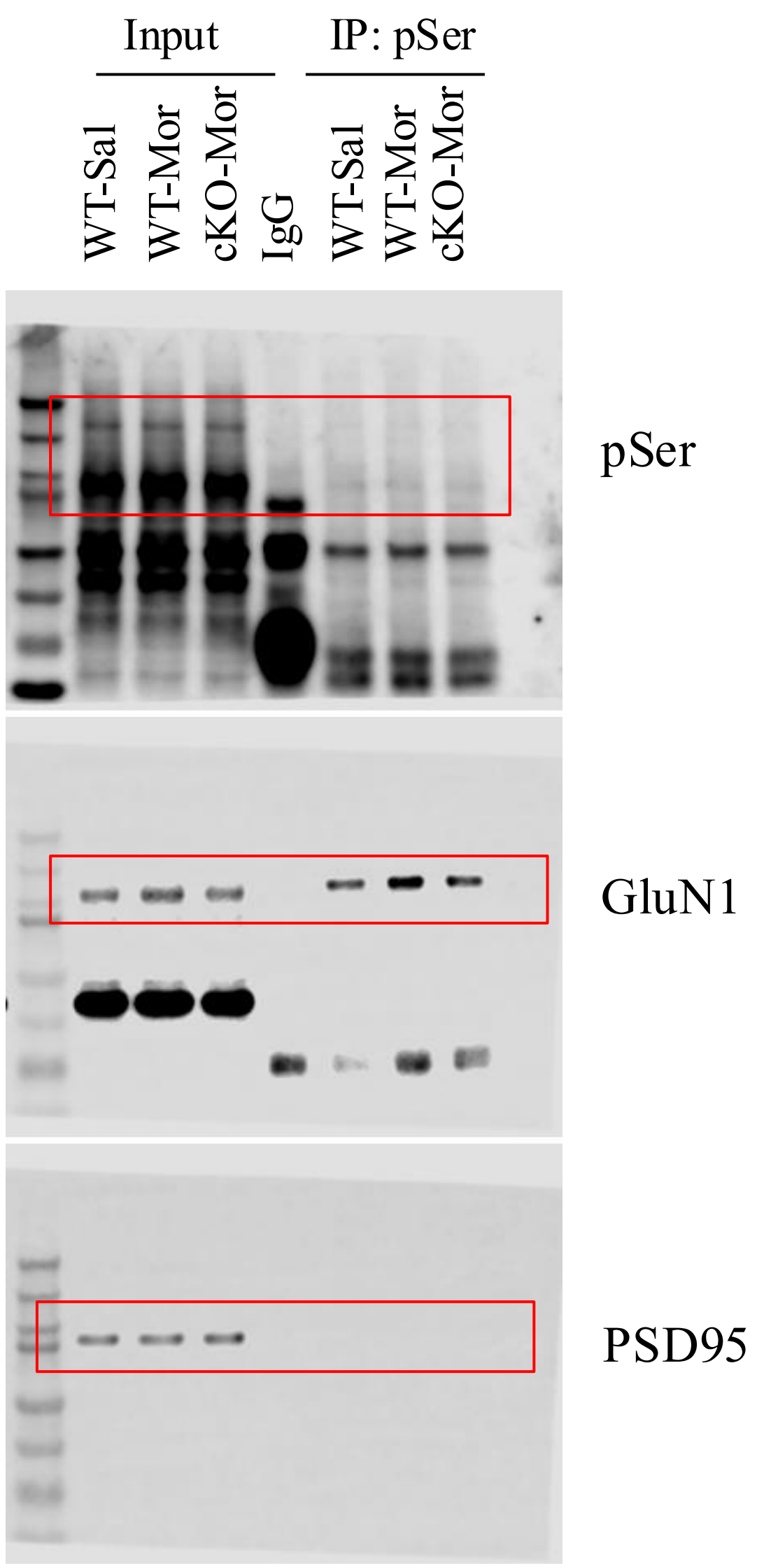

Input
IP: pSer
WT-Sal
WT-Mor
cKO-Mor
IgG
WT-Sal
WT-Mor
cKO-Mor
pSer
GluN1
PSD95

Fig. 9B

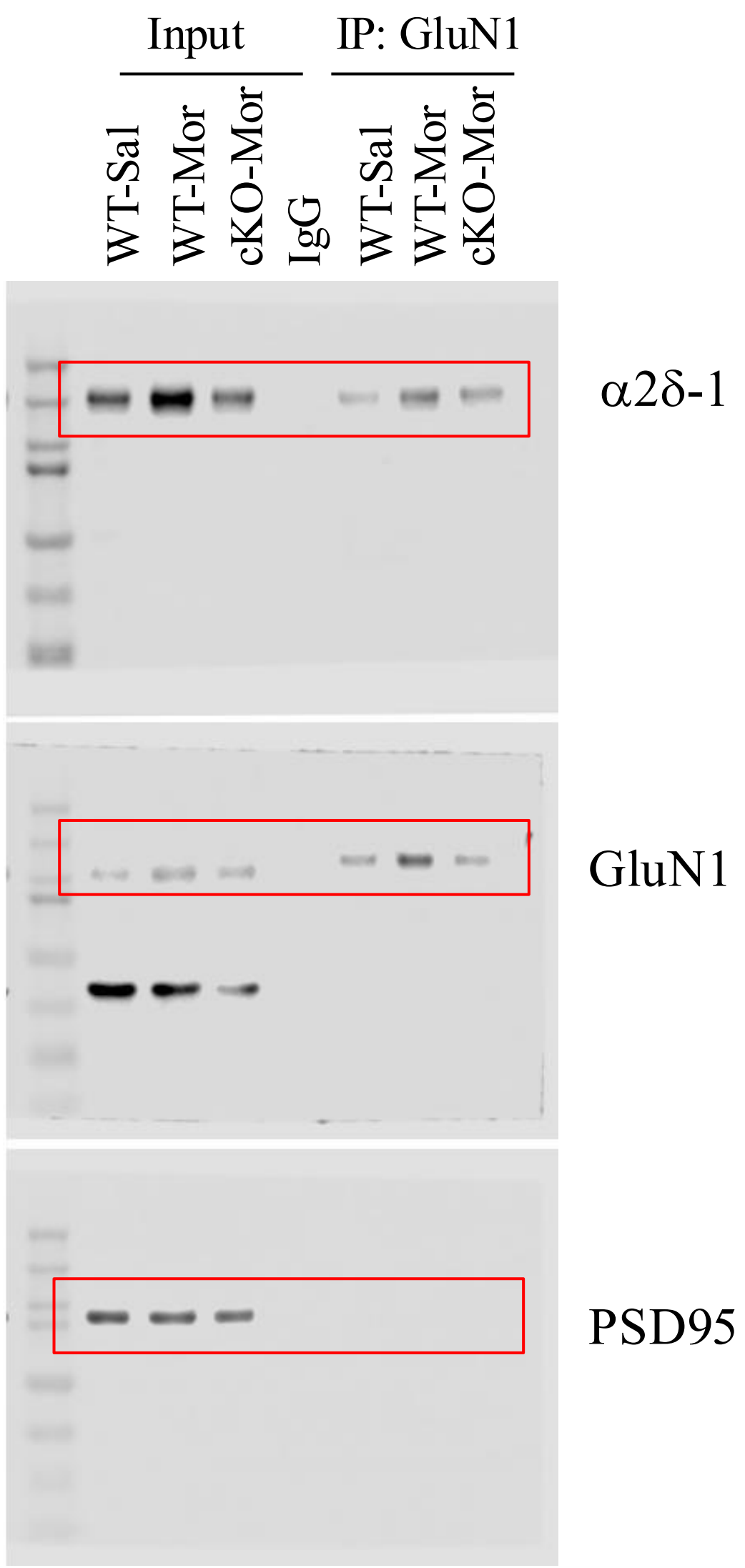

Input
IP: GluN1
WT-Sal
WT-Mor
cKO-Mor
IgG
WT-Sal
WT-Mor
cKO-Mor
α2δ-1
GluN1
PSD95

Fig. S1C DRG

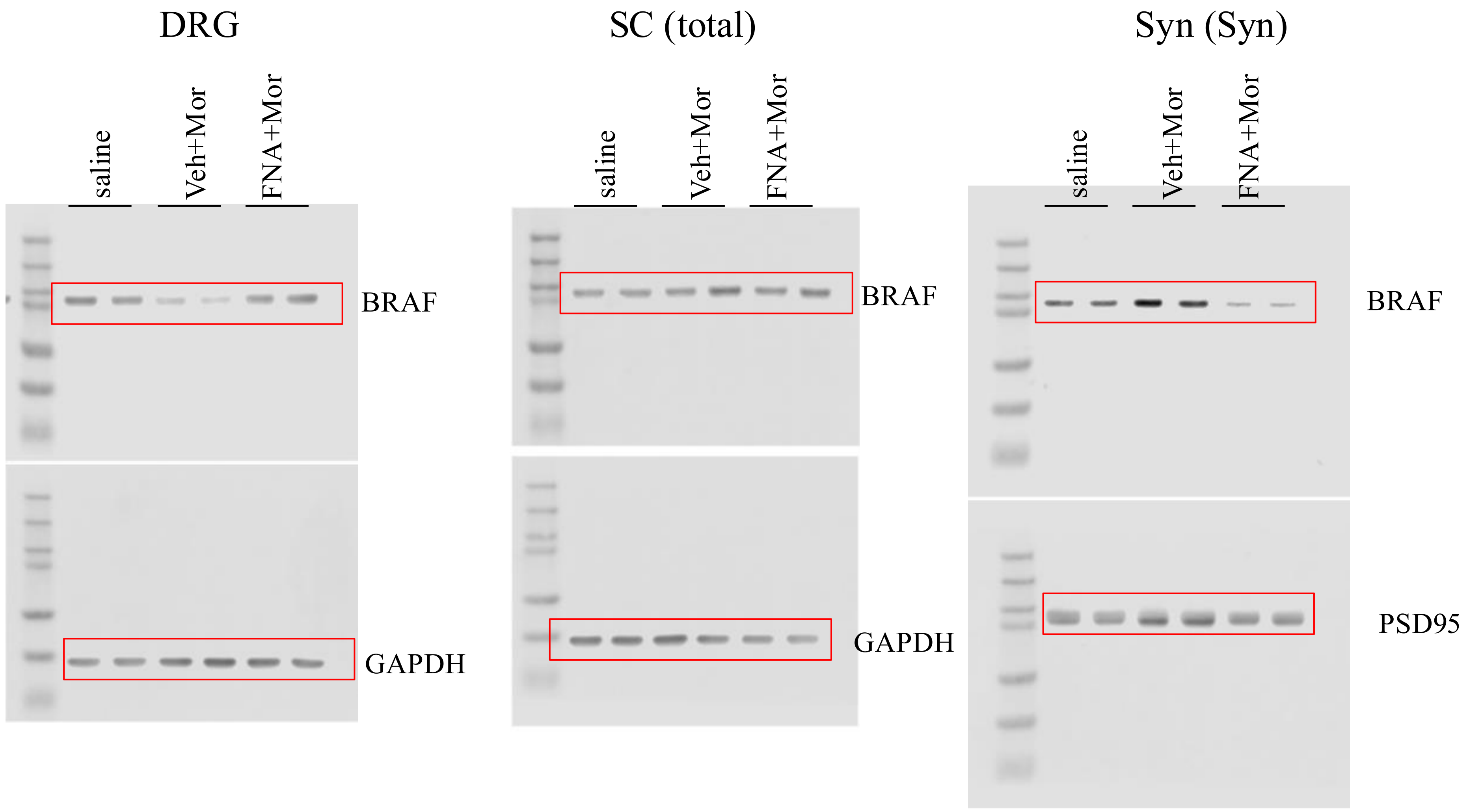
DRG
saline
Veh+Mor
FNA+Mor
BRAF
GAPDH
SC (total)
saline
Veh+Mor
FNA+Mor
BRAF
GAPDH
Syn (Syn)
saline
Veh+Mor
FNA+Mor
BRAF
PSD95

Fig. S2

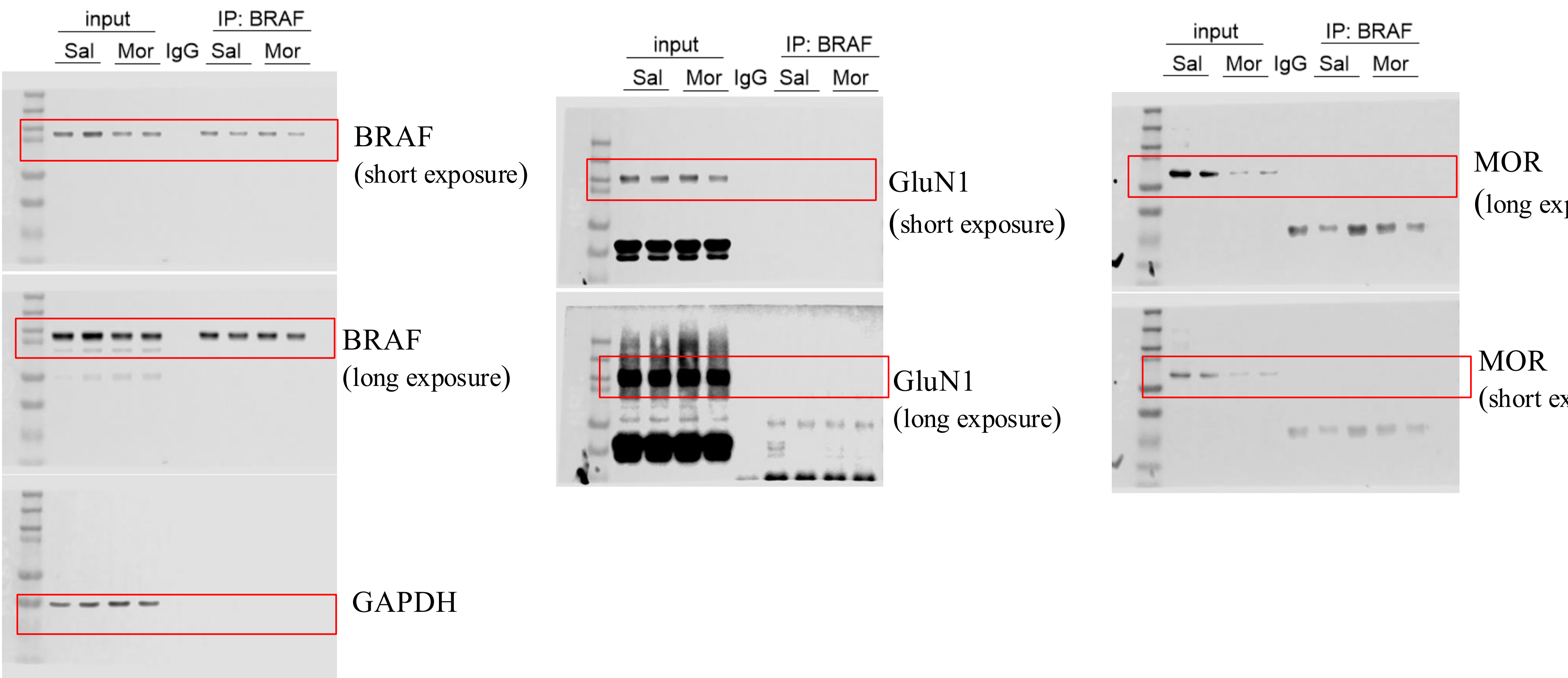

input
IP: BRAF
Sal
Mor
IgG
Sal
Mor
BRAF
(short exposure)
BRAF
(long exposure)
GAPDH
input
IP: BRAF
Sal
Mor
IgG
Sal
Mor
GluN1
(short exposure)
GluN1
(long exposure)
input
IP: BRAF
Sal
Mor
IgG
Sal
Mor
MOR
(long
MOR
(short

Fig. S3

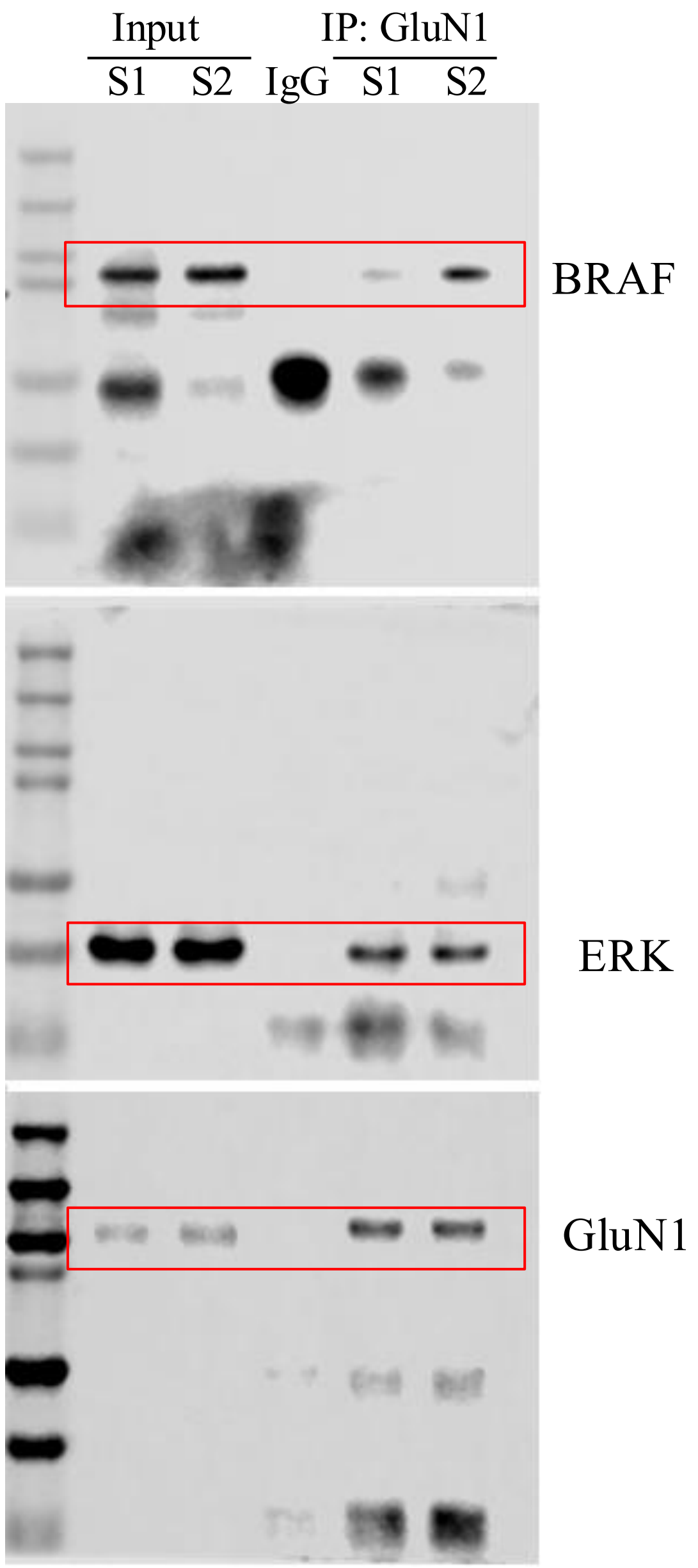

Fig. S4. Braf siRNA

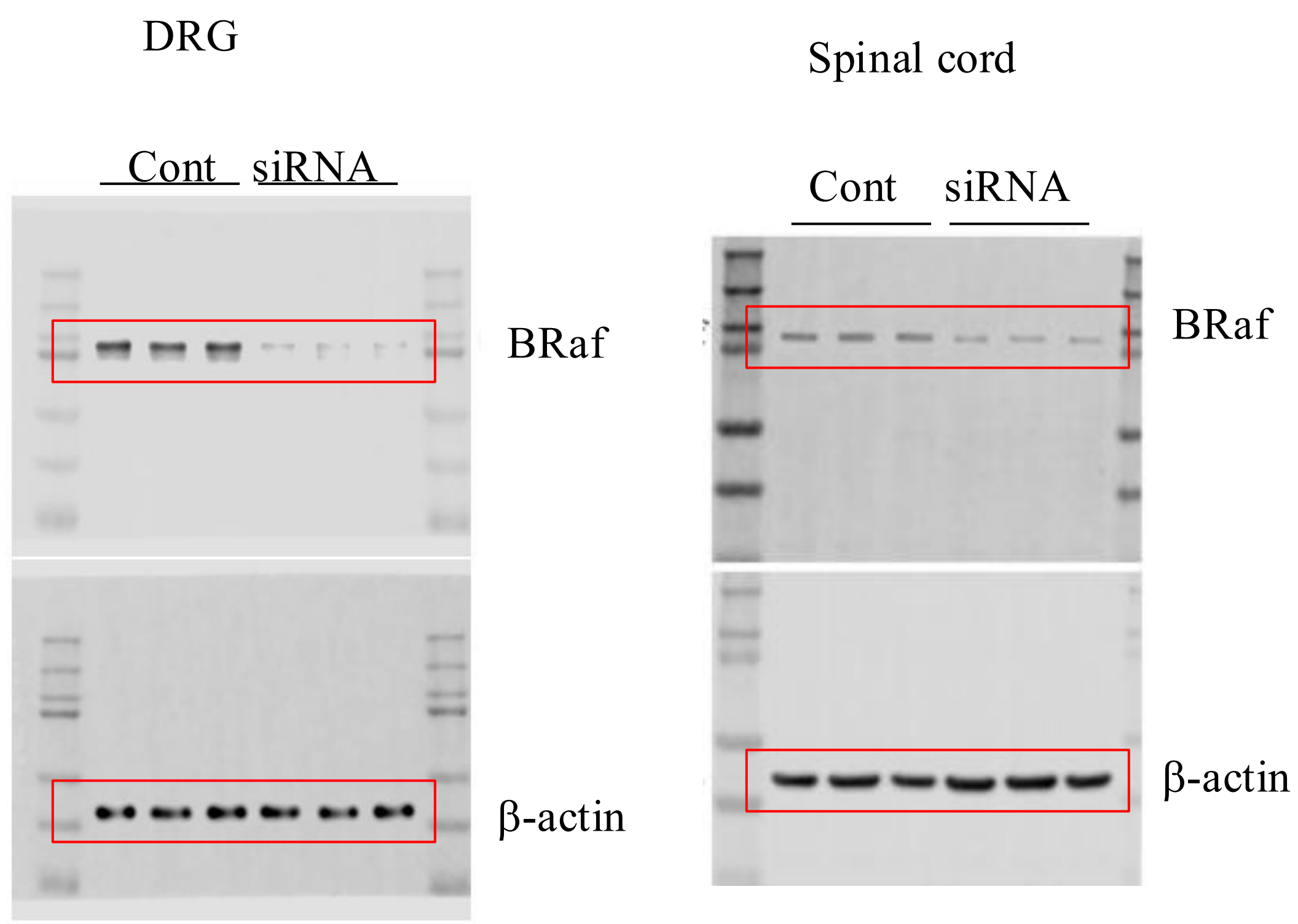

Supplement: Supplementary file 2 [file jci-136-201808-s069.pdf]
